# Busulfan-cyclophosphamide versus cyclophosphamide-busulfan as conditioning regimen before allogeneic hematopoietic cell transplantation: a prospective randomized trial

**DOI:** 10.1007/s00277-020-04312-y

**Published:** 2020-10-23

**Authors:** Claire Seydoux, Michael Medinger, Sabine Gerull, Joerg Halter, Dominik Heim, Yves Chalandon, Stavroula Masouridi Levrat, Urs Schanz, Gayathri Nair, Marc Ansari, Patrick Simon, Jakob R. Passweg, Nathan Cantoni

**Affiliations:** 1 Divisions of Hematology and Internal Medicine, Department of Medicine, University Hospital of Basel, Petersgraben 4, CH-4031 Basel, Switzerland; 2 Division of Hematology, Bone Marrow Transplant Unit, University Hospital of Geneva and Faculty of Medicine, University of Geneva, Geneva, Switzerland; 3 Department of Medical Oncology and Hematology, Stem-/Immune- cell-transplant Unit, University Hospital of Zurich, Zurich, Switzerland; 4 Department Women, Children and Adolescent, Unit of Oncology and Hematology Pediatric, University Hospital of Geneva, Geneva, Switzerland; 5 Cansearch Research Laboratory, Faculty of Medicine, University of Geneva, Geneva, Switzerland; 6 Clinical Trials Unit, Department of Clinical Research, Basel University, Basel, Switzerland; 7 Division of Oncology, Hematology and Transfusion Medicine, Kantonsspital Aarau, Aarau, Switzerland

**Keywords:** Busulfan, Conditioning regimen, Cyclophosphamide, Hematopoietic cell transplantation, Liver toxicity

## Abstract

Busulfan and cyclophosphamide (BuCy) is a frequently used myeloablative conditioning regimen for allogeneic hematopoietic cell transplantation (allo-HCT). Theoretical considerations and pharmacological data indicate that application of busulfan prior to subsequent cyclophosphamide (BuCy) may trigger liver toxicity. Reversing the order of application to cyclophosphamide-busulfan (CyBu) might be preferable, a hypothesis supported by animal data and retrospective studies. We performed a prospective randomized trial to determine impact of order of application of Bu and Cy before allo-HCT in 70 patients with hematological malignancy, 33 patients received BuCy and 37 CyBu for conditioning. In the short term, there were minimal differences in liver toxicity favoring CyBu over BuCy, significant only for alanine amino transferase at day 30 (*p* = 0.03). With longer follow-up at 4 years, non-relapse mortality (6% versus 27%, *p* = 0.05) was lower and survival (63% versus 43%, *p* = 0.06) was higher with CyBu compared to BuCy. Other outcomes, such as engraftment (*p* = 0.21), acute and chronic graft-versus-host disease (*p* = 0.40; 0.36), and relapse (*p* = 0.79), were similar in both groups. We prospectively show evidence that the order of application of Cy and Bu in myeloablative conditioning in allo-HCT patients has impact on outcome.

**Electronic supplementary material:**

The online version of this article (10.1007/s00277-020-04312-y) contains supplementary material, which is available to authorized users.

## Introduction

The combination of busulfan and cyclophosphamide (BuCy) is a frequently used established myeloablative conditioning regimen before allogeneic cell transplantation (allo-HCT). Antileukemic effects and myeloablative properties of this regimen in patients with hematological malignancies have been confirmed in multiple studies [1–4]. Interactions between busulfan (Bu) and cyclophosphamide (Cy) are well described [5, 6]; studies have shown that Bu affects the hepatic metabolism of Cy and may therefore increase liver toxicity when given in this order [7, 8]. The pathophysiology behind this interference is possibly a decrease in levels of glutathione, which is a major player in the breakdown of metabolites of Cy in the hepatocyte [9, 10]. Cy-induced toxicity has been associated with non-relapse mortality (NRM) and decreased survival in clinical studies [11]. The most feared hepatic complication is veno-occlusive disease/sinusoidal obstruction syndrome (VOD/SOS) described with the BuCy regimen [12–15]. As suspected in some retrospective clinical and animal studies, reversing the order of application of BuCy to CyBu may decrease liver toxicity without loss in antileukemic activity [6, 16–19]. In earlier years, when only oral Bu was available, the order of BuCy was preferred because of the emetogenic potential of Cy precluding administration of correct doses of Bu. With the introduction of intravenous Bu, associated with reduced liver complications and mortality [7], these considerations influencing the order of application no longer apply.

With this prospective randomized controlled trial (RCT), we aimed to test the impact of the order of application of BuCy versus CyBu prior to allo-HCT in patients with hematological malignancies.

## Patients and methods

### Study cohort and design

This is a prospective multicenter (University Hospitals Basel, Zurich, and Geneva) open label 1:1 RCT, comparing the order of application of busulfan followed by cyclophosphamide (BuCy, standard group) versus cyclophosphamide followed by busulfan (CyBu, experimental group) as myeloablative conditioning regimen prior to allo-HCT done between 2013 and 2017. The study was approved by the local ethics committee (EKNZ EKBB179/12), by Swissmedic (2012DR4164) and was registered with ClinicalTrials.gov as NCT01779882. Randomization was centrally at the Clinical Trial Unit (CTU) of the Basel University Hospital.

The primary endpoint, as defined in the protocol, was liver toxicity of BuCy versus CyBu at day 30 after allo-HCT, defined as abnormality in the levels of bilirubin, aspartate amino transferase (ASAT), alanine amino transferase (ALAT), gamma glutamyl transpeptidase (GGT), or alkaline phosphatase (AP). In addition, we used the NCI Common Terminology Criteria for Adverse Events (CTCAE version 4.0) grading of hepatic toxicity [20].

Secondary endpoints were the incidence and severity of VOD, incidence of other organ toxicity before day 30 and at day 100, incidence and severity of acute and chronic GvHD, and overall survival, relapse, and non-relapse mortality incidence at day 100 and at long-term follow-up.

Consenting and included patients were adults planned for myeloablative conditioning allo-HCT to treat acute myeloid leukemia (AML), chronic myeloid leukemia (CML), acute lymphoblastic leukemia (ALL), and myelodysplastic syndrome (MDS) or myeloproliferative neoplasm (MPN). They had an HLA-identical sibling or an allele (10/10) HLA-matched unrelated donor. Patients with preexisting active hepatitis or abnormal pretransplant liver function tests within 2 weeks prior to starting conditioning, such as bilirubin > 3 × upper limit of normal (ULN) or ASAT/ALAT > 5 × ULN, were excluded.

The inclusion goal was set at 72 patients (36 patients in each arm) to ensure 65 evaluable patients considering a drop-out rate of 10%, permitting a difference of 35% in detection of any abnormal liver values between the two groups on days 30 and 100. These assumptions, and hence the accrual goal, were derived from a previous retrospective study comparing CyBu with BuCy [17].

Patient baseline characteristics, such as sex, age, type, and stage of disease, EBMT risk score, hematopoietic cell transplantation-specific comorbidity index (HCT-CI) (Sorror), Karnofsky performance score (KPS), viral serology, specifically hepatitis A, B, and C (HAV, HBV, HCV), cytomegalovirus (CMV) and Ebstein-Barr-virus (EBV), prior autologous or allo-HCT, and transplant characteristics including donor type, sex, age and viral serology, stem cell source, and type of GvHD prophylaxis were recorded.

Liver function tests were measured on the day of enrollment and on days 0, 10, 20, 30, and 100, measuring levels of bilirubin, ASAT, ALAT, GGT, and AP. Outcomes, including time to neutrophil engraftment (defined as the first of 3 consecutive days of an absolute neutrophil count exceeding 0.5 × 10^9^/L), cumulative incidence of VOD, aGvHD, and cGvHD, and grade and treatment, were measured. Survival was defined as time from transplantation to death or last follow-up, relapse was defined as hematologic relapse after allo-HCT, and NRM was death without prior relapse.

### Definitions

VOD was defined using the modified Seattle criteria [21] with the occurrence of two of the following events within 20 days of transplantation: hyperbilirubinemia (total serum bilirubin > 34.2 μmol/L (2 mg/dL)), hepatomegaly or right upper quadrant pain of liver origin, and sudden weight gain (> 2% of baseline body weight) because of fluid accumulation.

Acute and chronic GvHD were diagnosed clinically and confirmed histologically when possible. Grading and staging used consensus classifications for acute and chronic GvHD [22, 23]. Acute GvHD was defined as clinically relevant with grade ≥ II.

### Treatment

The standard group received the regular conditioning regimen: i.v. Bu (0.8 mg/kg every 6 h, as a 2-h infusion in NaCl 0.9% for a final concentration of 0.5 mg/mL; total 16 doses) from days − 8 to − 4, followed by i.v. Cy (60 mg/kg in 5% glucose) on days − 3 and − 2. Busulfan was started on the evening of day − 8 such that the last dose was given at a time interval of at least 24 h before the first cyclophosphamide dose [8]. The experimental group received i.v. Cy on days − 8 and − 7, and i.v. Bu from days − 5 to − 2, in the same doses but in reversed order (supplementary file study protocol).

Busulfan pharmacokinetic dose adjustment was performed from the fifth dose onward to achieve a target Css from 800 to 1000 ng/mL according to the centers’ guidelines [24]. VOD prophylaxis consisted of intravenous heparin 5000 IU/24 h and ursodeoxycholic acid 250 mg po 3 times daily in two of the centers; one center (7 patients included) did not use VOD prophylaxis. Patients with proven or highly probable VOD were treated with defibrotide i.v. 6.25 mg/kg every 6 h until resolution.

GvHD prophylaxis consisted of cyclosporine A (CsA) (3 mg/kg bw/day i.v.; starting day − 3 over 6 h adjusted to blood trough levels targeting 150-200 μg/L) and methotrexate (MTX) (15 mg/m^2^ i.v. day + 1; 10 mg/m^2^ day + 3 and day + 6). In addition, for HCT from unrelated donors and in one center for matched related donors ≥ 40 years [25], ATG (*n* = 45) or alemtuzumab (*n* = 3) was used (Table 1). CsA was tapered and discontinued 6 months post-transplant.

**Table 1:** Patient, disease, and transplant characteristics

	CyBu *n* = 37	BuCy *n* = 33	*p* value
Age (median, years; range)	47 (21-62)	46 (20-65)	0.49
Gender (male, %)	25 (68)	15 (46)	0.06
Donor/recipient gender			
Female/male (*n*, %)	9 (24)	5 (15)	0.26
Other (*n*, %)	28 (76)	28 (85)	
Disease			
AML (*n*, %)	28 (76)	24 (73)	0.70
MDS/MPN (*n*, %)	7 (19)	7 (21)	
CML (*n*, %)	2 (5)	1 (3)	
ALL (*n*, %)	0	1 (3)	
Prior HCT			
Allogenic (*n*, %)	1 (3)	1 (3)	0.94
Autologous (*n*, %)	2 (5)	5 (15)	0.18
Disease status at HCT			
CR or chronic phase 1 (*n*, %)	27 (73)	22 (67)	0.47
2.CR or never treated (*n*, %)	4 (11)	7 (21)	
No CR (*n*, %)	6 (16)	4 (12)	
Stem cell source			
Peripheral blood (*n*, %)	37 (100)	31 ( 94)	0.13
Bone marrow (*n*, %)	0 (0)	2 (6)	
Donor			
HLA-identical sibling (*n*, %)	19 (51)	15 (46)	0.62
HLA-matched unrelated (*n*, %)	18 (49)	18 (54)	
Donor age (median, years; range)	43 (20-68)	40 (20-61)	0.23
Donor/recipient CMV status			0.23
Neg/pos (*n*, %)	2 (5)	7 (21)	
Other (n, %)	35 (95)	26 (79)	
GvHD prophylaxis			
ATG or T-depletion (*n*, %)	27 (73)	21 (64)	0.33
CyA + MTX (*n*, %)	6 (16)	10 (30)	
CyA alone (*n*, %)	4 (11)	2 (6)	
Center			
A	29 (78)	27 (82)	0.42
B	4 (11)	5 (15)	
C	4 (11)	1 (3)	
HCT-CI			
0	20 (54)	15 (46)	0.091
1-2	17 (46)	14 (42)	
> 2	0 (0)	4 (6)	
KPS^1^			
90-100	8 (22)	2(6)	0.06
< 80	29 (78)	31 (94)	

*allo-HCT* allogeneic cell transplantation; *CMV* cytomegalovirus; *AML* acute myeloid leukemia; *MDS* myelodysplastic syndrome; *MPN* myeloproliferative neoplasm; *CML* chronic myeloid leukemia; *ALL* acute lymphoblastic leukemia; *CR* complete remission; *2.CR* second complete remission; *EBMT* European Group for Blood and Marrow Transplantation; *aGvHD* acute graft-versus-host-disease; *ATG* anti-thymocyte globulin; *CyA* cyclosporine A; *MTX* methotrexate; *HCT-CI* hematopoietic cell transplantation-specific comorbidity index; *KPS* Karnofsky performance status

^1^One death before allo-HCT in CyBu of multiple organ failure from toxic megacolon

Supportive care was as per institutional guidelines and included trimethoprim/sulfamethoxazole, fluconazole, and valacyclovir. Lorazepam was given before Bu to reduce central nervous system adverse reactions.

### Statistical analysis

Data are reported as mean or median with standard deviation (SD) or with interquartile range (IQR) as appropriate. Differences among groups were analyzed using Chi-square or Fisher’s exact test for categorical variables, and Student’s *t* or Mann-Whitney *U* test for continuous variables, depending on data distributions. Variables significantly associated with outcome in univariate analysis were included in multivariate Cox proportional hazards regression models. All *p* values were two-sided and statistical significance was determined by a *p* value < 0.05. Cumulative incidence function of NRM and relapse was performed using death of other causes as competing event, with Fine and Gray to test for differences. The Kaplan-Meier estimator and the log-rank test were used for overall survival. Statistical analysis was performed using the SPSS (version 22; IBM, Chicago, IL, USA) and STATA SE (version 15; StataCorp LLC, College Station, TX, USA) software.

## Results

### Patient baseline characteristics

Of 114 patients screened, 72 were eligible. Screening failures were mainly because of preexisting liver abnormalities. A total of 72 patients were randomized from 2013 to 2017, and 2 were excluded because of delayed identification of preexisting hepatitis and for donor problems leaving 70 evaluable patients in the trial. Of the 70 evaluable patients, 56 (80%) were treated in center A, 9 (13%) in center B, and 5 (7%) in center C (Table 1). Median age was 47 years and 57% were male. Transplants were from HLA-identical siblings (*n* = 34, 49%) or from matched unrelated donors (*n* = 36, 51%). Nine patients had a prior HCT (2 allogeneic and 7 autologous). Median follow-up of surviving patients was 1092 (IQR 730-1364) days. Disease was AML (*n* = 52, 74%), MDS/MPN (*n* = 14, 20%), CML (*n* = 3, 4%), and ALL (*n* = 1, 2%). Early disease stage (CR (complete remission) or first chronic phase) was in 49 (70%) patients, intermediate (second CR or treated upfront) in 11 (16%), and advanced (no CR) in 10 (14%) patients. A total of 33 patients were randomized to the standard group (BuCy) and 37 to the experimental group (CyBu). Distribution of patient, disease, and transplant characteristics is shown in Table 1 and did not differ significantly among groups.

### Liver toxicity and outcomes

Liver function tests measured as bilirubin, ASAT, ALAT, GGT, and AP were not different between groups at baseline (Table 2). The only significant difference was higher levels of ALAT (median 27 versus 22 IU/L, *p* = 0.03) in the BuCy as compared to the CyBu group on day 30. All other liver function tests did not differ among groups; on day 100, no significant differences were seen.

**Table 2:** Liver toxicity

	CyBu (*n* = 37)	BuCy (*n* = 33)	*p* value
Day 30			
ASAT (median; range)	27 (7-64)	30 (12-882)	0.09
ALAT (median; range)	22 (8-89)	27 (12-676)	*0.03*
GGT (median; range)	47 (19-261)	67 (22-876)	0.08
AP (median; range)	69 (23-177)	80 (29-325)	0.16
Bilirubin (median; range)	10 (4-53)	10 (3-172)	0.71
Day 100			
ASAT (median; range)	29 (17-74)	32 (10-78)	0.45
ALAT (median; range)	31 (12-93)	31 (5-136)	0.76
GGT (median; range)	38 (14-804)	51 (17-364)	0.20
AP (median; range)	68 (37-274)	67 (25-208)	0.96
Bilirubin (median; range)	9 (3-28)	7 (3-19)	0.79
CTCAE grade day 30			
Grade 0 (*n*, %)	19 (51)	12 (36)	0.21
Grade ≥ 1 (*n*, %)	18 (49)	21 (64)	
CTCAE grade day 100			
Grade 0 (*n*, %)	23 (62)	16 (48)	0.25
Grade ≥ 1 (*n*, %)	14 (38)	17 (52)	
CTCAE maximum grade			
Grade 0 (*n*, %)	15 (40)	7 (21)	0.08
Grade *>* 1 (*n*, %)	22 (60)	26 (79)	

*ASAT* aspartate amino transferase; *ALAT* alanine amino transferase; *GGT* gamma glutamyl transpeptidase; *AP* alkaline phosphatase

Slightly more patients in the BuCy as compared to the CyBu group had any grade of CTCAE liver toxicity criteria on day 30 or day 100 (mostly grade 1 toxicity). Even when combining all CTCAE toxicity criteria, differences did not reach statistical significance (*p* = 0.08).

**Fig. 1:**
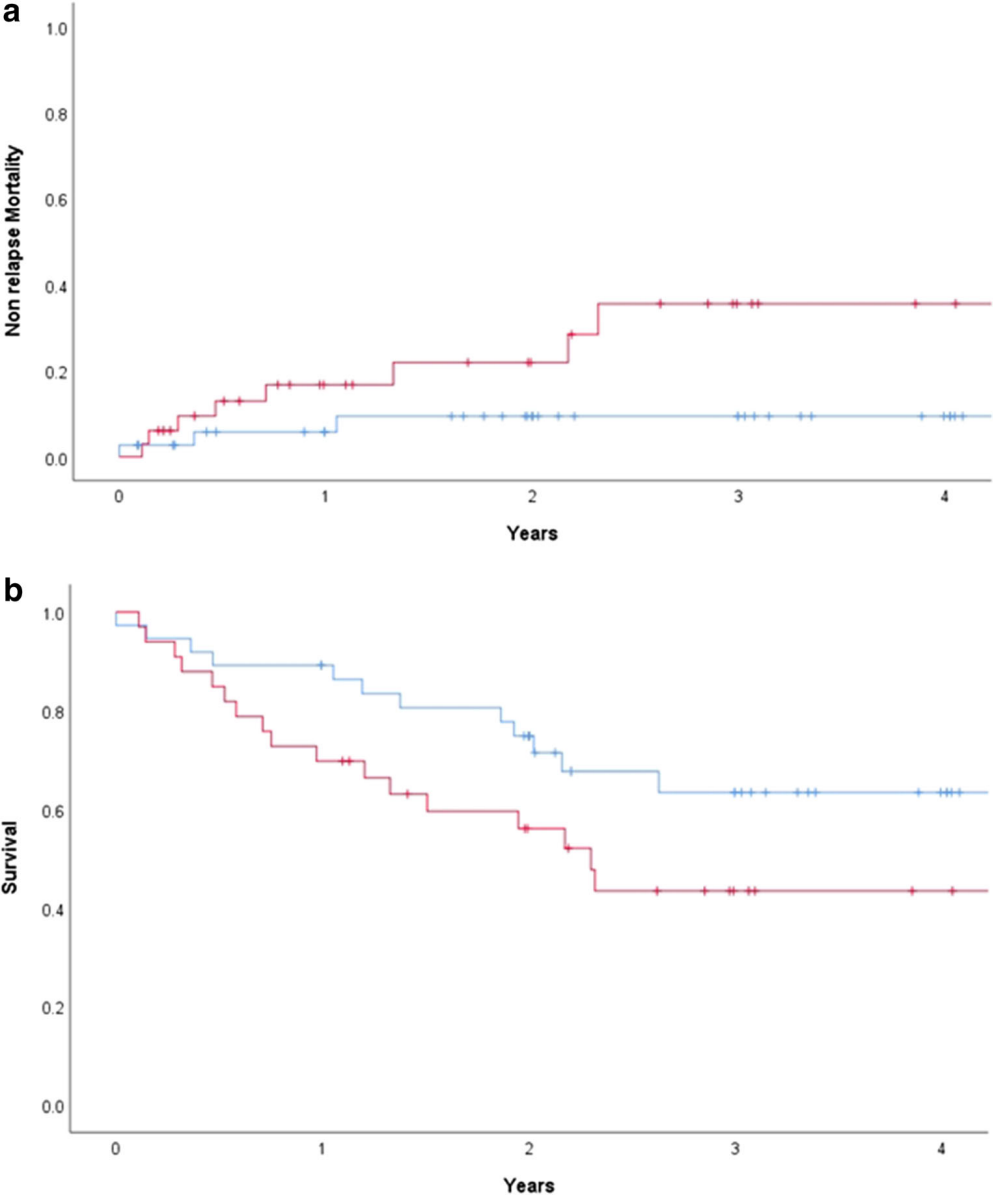
**a** Non-relapse mortality (CyBu in blue, BuCy in red). **b** Survival (CyBu in blue, BuCy in red)

One fatal VOD episode occurred in the BuCy group versus none with CyBu; the frequency of patients fulfilling at least one of the predefined VOD criteria, but not being formally diagnosed with VOD, was significantly higher in BuCy versus CyBu groups (17 versus 10 patients; *p* = 0.05). Median AUC and Css showed no significant differences between the two groups: AUC (1126 (630-1595) versus 1006 (582-2477) μmol/L*min, *p* = 0.42) and Css (793 (430-1091) versus 689 (398-1693) ng/mL, *p* = 0.54) in BuCy versus CyBu in 66 of 70 patients. The cumulative incidence of NRM at 4 years was higher in the BuCy compared to CyBu (27 (15-49)% versus 6 [2–22]%; *p* = 0.049, Fig. 1a). Cause of death in patients with NRM did not differ among groups (*p* = 0.32, see Table 3). The survival probability at 4 years in the BuCy group tended to be lower (43 ± 19% versus 63 ± 17%; *p* = 0.06, Fig. 1b).

**Table 3:** Patient outcomes and univariate analysis

	CyBu (*n* = 37)	BuCy (*n* = 33)	*p* value
Engraftment (mean days, 95% CI)	16 (15-17)	15 (14-16)	0.27
VOD between day 0 and day 30 (*n*, %)	0	1 (3)	
No criterion (*n*, %)	27 (73)	16 (48)	*0.05*
1 or more criteria^1^ (*n*, %)	10 (27)	17 (52)	
aGvHD grade ≥ II (%, 95% CI)	14 (6-31)	27 (16-48)	0.20
cGvHD (at 2 years, %, 95% CI)	52 (38-72)	39 (25-60)	0.36
Cause of death			
Relapse (*n*, %)	9 (75)	10 (56)	0.32
GvHD (*n*, %)	1 (8)	2 (11)	
VOD (*n*, %)	0	1 (6)	
Infection (*n*, %)	0	4 (22)	
Toxicity (*n*, %)	2 (17)	1 (6)	
Long-term outcome			
Relapse/progression (at 4 years, %, 95% CI)	34 (22-55)	34 (21-55)	0.79
Non-relapse mortality (at 4 years, %, 95% CI)	6 (2-22)	27 (15-49)	0.049
Survival (at 4 years, %, 95% CI)	63 (46-80)	43 (24-62)	0.06

*VOD* veno-occlusive disease; *HCT* hematopoietic cell transplantation; *CI* confidence interval; *aGvHD* acute graft-versus-host disease; *cGvHD* chronic graft-versus-host disease

^1^Including hyperbilirubinemia > 34 mmol/L, painful hepatomegaly, ascites

In multivariate analysis adjusting for Karnofsky performance score and hematopoietic cell transplantation-specific comorbidity index, RR of death in the BuCy group compared to CyBu was 2.270 (0.98-5.27), *p* = 0.056 and corresponding risk of NRM was 4.76 (1.01-22.42), *p* = 0.049 confirming results of univariate analysis.

Median time to engraftment was similar in both groups (15 [14–16] days versus 16 [15–17] days, *p* = 0.27). The cumulative incidence for aGvHD grade ≥ II and cGvHD was similar in BuCy (27 (16-48)% and 39 (25-60)%) compared to CyBu 14 ( [6–31]%; and 52 (38-72)% respectively; *p* = 0.20 for aGvHD and *p* = 0.36 for cGvHD). Relapse at 4 years was observed in a total of 24 patients (12 in BuCy and 12 in CyBu). In both groups, the cumulative incidence of relapse was similar (34 (21-55)% versus 34 (22-55)%; *p* = 0.79).

## Discussion

In this RCT, we compared the order of application of busulfan and cyclophosphamide for myeloablative conditioning before allogeneic cell transplantation in patients with hematological malignancy. Results of this trial support prior hypotheses that the order of application of Cy and Bu may have an impact on short- and long-term toxicity and outcome after allo-HCT. Our study is in line with previous retrospective and animal studies [6, 7, 16–18], by showing a somewhat lower early hepatic toxicity and a lower NRM (*p* = 0.49) at long-term follow-up after transplantation with CyBu compared to BuCy.

Among two retrospective studies, Cantoni et al. described increased liver toxicity at day 30, + higher VOD incidence and higher NRM in BuCy patients compared to CyBu [17]. Rezvani et al. conducted a similar study comparing CyBu to historic BuCy controls, reporting a lower incidence of VOD and a decreased day 100 mortality with CyBu in patients with myelofibrosis. In patients with AML or MDS, NRM differences were not significant [19]. The study discussed here included mainly patients with AML, the study by Rezvani et al., mainly MDS/MPN possibly explaining some differences. As incidence of acute and chronic GvHD and relapse was similar when comparing the CyBu to the BuCy arm in our study, the difference in NRM is not explained by the immunological complication of GvHD or by an impact on malignancy. The difference in long-term NRM is not fully explained by small differences in short-term hepatic toxicity.

Busulfan followed by cyclophosphamide (mostly in that order) is one of the most commonly used myeloablative regimens before allo-HCT; e.g., in patients with AML in the same time period as this trial (2013-2017), 41% of patients reported to the EBMT registry had BuCy as their conditioning regimen (personal communication, M. Labopin). Hassan et al. [8] had shown in a study that the time interval between busulfan and cyclophosphamide was of importance for toxicity. In our trial, this time interval was respected meticulously. In the study by Hassan et al., busulfan had been administered orally and not intravenously.

The hepatotoxicity of BuCy regimen is well described [12–14, 26]; one of the pathomechanism of the hepatotoxicity of Bu and Cy is the depletion in glutathione levels, which plays a central role in the metabolism of the toxic metabolites of Cy in hepatocytes [9, 10]. Glutathione depletion as a mechanism of hepatic toxicity had been studied extensively [27–29]. The precise pathomechanism remains speculative as we do not have data on glutathione metabolism and other explanations are possible.

While the incidence of VOD has decreased over the years, mortality of established VOD remains high [30]. It is known that inter-individual discrepancy of pharmacokinetics of busulfan impacts on toxicity outcomes [31–33]. The introduction of busulfan therapeutic drug monitoring between 2000 and 2010 led to a reduction in adverse outcomes regarding liver toxicity, highlighted by a reduction in the VOD incidence from 15% in the late 1990s to 3% after 2010 after dose adjustment of busulfan during this time period [26, 31]. In our trial, the incidence of severe VOD is 1.4% (one case in 70 patients) and this is comparable to the literature, where incidence of 2-5% has been described [26, 34]. We have to take into account that patients with preexisting liver problems were excluded. Furthermore, when using VOD criteria as defined by Mohty et al. [35], more patients in the BuCy arm had fulfilled at least one criterion than the CyBu arm.

Despite the power of a prospective randomized trial, we acknowledge limitations of this trial: Sample size is limited, and the clinical trial unit did not allow for randomization of a larger patient number, given published differences in retrospective studies [18]. Screening failure was 39% excluding patients with preexisting liver abnormalities, thus assuring the inclusion of patients with normal liver function, best suited to test the study hypothesis. Patient accrual was low because of a general tendency to increasingly use reduced intensity regimens. Randomization was not blinded; however, bias in measuring liver function tests or NRM is unlikely. Furthermore, we saw some heterogeneity based on center’s specific transplant procedures, such as GvHD and other prophylaxis (e.g., ATG use to prevent GvHD, heparin use to prevent VOD), and in diseases of patients randomized.

We found some limited differences in the hepatic toxicity, although the differences are not large and we do not know if they are clinically meaningful. Factors other than the conditioning regimen may impact liver function tests early after HCT. Differences in long-term survival and NRM had not been pre-specified in the study protocol. Causes of death did not differ significantly between groups. It appears that infectious death is more frequent in the ByCy group. Of the 4 infectious deaths, one patient had ATG, one had alemtuzumab, and two had no T cell depletion. Therefore, T cell depletion does not appear to explain small differences in infectious mortality.

## Conclusions

In conclusion, we demonstrate that order of application of cyclophosphamide and busulfan may influence outcome when used in myeloablative conditioning for hematological malignancy. Additional data need to be generated on pharmacology of drugs used in conditioning to achieve safer individualized chemotherapeutic treatment.

## Electronic supplementary material

ESM 1 (PDF 1551 kb)

## Data Availability

The datasets generated during and/or analyzed during the current study are available from the corresponding author on reasonable request.
