# Supplementary material for: Busulfan-cyclophosphamide versus cyclophosphamide-busulfan as conditioning regimen before allogeneic hematopoietic cell transplantation: a prospective randomized trial
Source: Ann Hematol. 2020 Oct 23;100(1):209–16. doi: 10.1007/s00277-020-04312-y (PMC7782401; doi:10.1007/s00277-020-04312-y)
Supplement: Supplementary file 1 — (PDF 1551 kb) [file 277_2020_4312_MOESM1_ESM.pdf]

Universitätsspital Basel

BUCYBU study - Cyclophosphamide-Busulfan versus Busulfan-Cyclophosphamide as Conditioning Regimen before Allogeneic Hematopoietic Stem Cell Transplantation for Leukemia: a Prospective Randomized Study to Assess Liver Toxicity

PROTOCOL

Version 5.0, August 2nd , 2013

**Trial Chairperson**:	Nathan Cantoni, MD<br>Division of Hematology, University Clinic of Medicine<br>Kantonsspital Aarau, 5001 Aarau, Switzerland
**Co-Chairperson:**	Sabine Gerull, MD<br>Division of Hematology<br>University Hospital Basel, 4031 Basel, Switzerland
**Steering Committee:**	Nathan Cantoni, Aarau<br>Jakob Passweg, Basel<br>Sabine Gerull, Basel<br>Urs Schanz, Zürich<br>Yves Chalandon, Geneva<br>Marc Ansari, Geneva
Data Safety Monitoring Committee:	Mario Bargetzi, Aarau<br>Tayfun Güngör, Zürich<br>Thomas Lehmann, St. Gallen<br>Philipp Schütz, Aarau
**Sponsor:**	University Hospital Basel (UHBS), Switzerland
**Study drugs:**	Cyclophosphamide (Endoxan®), Baxter SA, Volketswil,<br>Busulfan (Busilvex®), Robapharm SA, Allschwil
**Datamanagement:**	Clinical Trial Unit (CTU) Basel<br>Schanzenstrasse 55 (BH 3)<br>4031 Basel, Switzerland
**SAE notification:**	CTU Basel
**Statistician:**	Thomas Fabbro, PhD (CTU Basel)
**Protocol version:**	Version 5.0, 02.08.2013

1 PROTOCOL SIGNATURE PAGE

BUCYBU study - Cyclophosphamide-Busulfan versus Busulfan-Cyclophosphamide as Conditioning Regimen before Allogeneic Hematopoietic Stem Cell Transplantation for Leukemia: a Prospective Randomized Study to Assess Liver Toxicity

The protocol was accepted by the steering committee. The final protocol is dated 02.08.2013.

Sponsor Representative:

Name: Jakob Passweg

Date: 7/8/13 Signature: ____________

Trial Chairperson:

Name: Nathan Cantoni

Date: 02.08.2013 Signature: N. Cantoni

Trial Co-Chairperson:

Name: Sabine Gerull

Date: 08.08.2013 Signature: S. Gerull

Trial Statistician:

Name: Thomas Fabbro

Date: 2013-08-06 Signature: ____________

2 ABBREVIATIONS

AE	Adverse event
Ag	Antigen
ALT	Alanine amino transferase
AML	Acute myeloid leukemia
ANC	Absolute neutrophil count
AP	Alkaline phosphatase
ASR	All-subjects-randomized
AST	Aspartate amino transferase
AUC	Area under the curve
BER	Base excision repair
BU	Busulfan
CML	Chronic myeloid leukemia
CR	Complete remission
CRA	Clinical Research Associate
CRF	Case report form(s)
CT	Computed tomography
CTCAE	Common terminology criteria for adverse events
CTU	Clinical Trial Unit
CV	Curriculum vitae
CY	Cyclophosphamide
CYP450	Cytochrome P450
DBS	Dried blood spot
DSMC	Data Safety Monitoring Committee
DNA	Deoxyribonucleic acid
EBMT	European Group for Blood & Marrow Transplantation
GCP	Good clinical practice
GGT	Gamma glutamyl transpeptidase
GST	Glutathione-S-Transferase
GVHD	Graft-versus-host disease
HIV	Human immunodeficiency virus
HLA	Human leucocyte antigen
HSCT	Hematopoietic stem cell transplantation
HUG	University Hospital Geneva
ICH	International Conference on Harmonisation

ISF	Investigator Site File
ITT	Intention to treat
i.v.	Intravenous
MDS	Myelodysplastic syndrome
MPN	Myeloproliferative neoplasia
NCI	National Cancer Institute
OClin	Ordonnance sur les essais cliniques de produits thérapeutiques
PI	Principal investigator
PK	Pharmacokinetics
PP	Per protocol
SADR	Serious adverse drug reaction
SAE	Serious adverse event
SBST	Swiss Blood and Marrow Stem Cell Transplant Group
SDV	Source data verification
SUSAR	Suspected unexpected serious adverse reaction
TBI	Total body irradiation
TDM	Therapeutic drug monitoring
TMF	Trial master file
UHBS	University Hospital Basel
ULN	Upper limit of normal
UPN	Unique patient number
USZ	University Hospital Zürich
VKlin	Verordnung über klinische Versuche mit Heilmitteln
VOD	Veno-occlusive disease
WBC	Whole blood count
WHO	World health organization

3 TABLE OF CONTENTS

PROTOCOL 1
1 PROTOCOL SIGNATURE PAGE 3
2 ABBREVIATIONS 4
3 TABLE OF CONTENTS 6
4 TRIAL OVERVIEW (SYNOPSIS) 10
4.1 Objectives 10
4.2 Primary endpoint 10
4.3 Secondary endpoints 10
4.4 Trial design 10
4.5 Selection of patients (most important criteria) 10
4.6 Trial duration 10
4.7 Statistical considerations 10
4.8 Trial Treatment 11
5 SCHEME OF THE TRIAL 11
6 INTRODUCTION AND BACKGROUND 12
6.1 Therapy background 12
6.2 Busulfan 12
6.2.1 Mechanism of action(8) 12
6.2.2 Absorption and bioavailability(8) 13
6.2.3 Distribution(8) 13
6.2.4 Metabolism(8) 13
6.2.5 Elimination(8) 14
6.2.6 Drug-related adverse events(8) 14
6.3 Cyclophosphamide 14
6.3.1 Metabolism and mechanism of action(9) 14
6.3.2 Distribution(9) 15
6.3.3 Elimination(9) 15
6.3.4 Drug-related adverse events(9) 15
6.4 Rationale for performing the trial 15
7 TRANSLATIONAL RESEARCH PROJECTS: BACKGROUND & METHODS 16
7.1 Cytokines profiling 16
7.2 Pharmakogenomics (Polymorphisms analysis) 16
7.3 alpha GST levels and glutathione analysis 17
7.4 Expression analysis of BER and GST pathway genes and their influence on outcome to treatment 18
8 OBJECTIVES AND ENDPOINTS 18
8.1 Study Aim 18
8.2 Primary endpoint 18
8.3 Secondary endpoints 18
8.4 Additional research questions 19
8.4.1 Cytokines measurement 19
8.4.2 Pharmacogenomics (polymorphisms) 19
9 TRIAL DESIGN, DURATION AND TERMINATION 19
10 SELECTION OF PATIENTS 20
10.1 Patient population 20
10.2 Inclusion criteria 20
10.3 Exclusion criteria 20

10.4 Enrollment 20
10.5 After enrollment 21
10.6 Randomization 21
10.7 Stratification 21

11 TRIAL TREATMENT 21
11.1 Therapy schedules 21
11.2 Handling, preparation and administration of CY and BU 22
11.3 Allogeneic HSCT 23
11.4 Supportive care 23

12 EVALUATIONS AND INVESTIGATIONS BEFORE, DURING AND AFTER TRIAL 23
12.1 Pretreatment evaluations and procedures 23
12.2 Evaluations to be performed within 21 days before treatment 23
12.3 Evaluations prior to start of the conditioning regimen 23
12.4 Evaluations during trial 24
12.5 Evaluations after treatment 24
12.6 Sampling for pharmacogenomics, and cytokines profiling 24

13 SAFETY 25
13.1 Adverse events: Definitions 25
13.1.1 Adverse Event (AE): 25
13.1.2 Adverse Reaction (AR): 25
13.1.3 Serious Adverse Event (SAE) or Serious Adverse Drug Reaction (SADR): 25
13.1.4 Suspected Unexpected Serious Adverse Reaction (SUSAR): 26
13.2 Causality assessment of adverse events 26
13.3 Handling of adverse events 26
13.3.1 Medical follow-up of adverse events 27
13.3.2 Data Safety Monitoring Committee (DSMC) 27
13.3.3 SAE coding 28
13.3.4 SAE and SUSAR reporting 28
13.3.5 Annual safety report 28
13.3.6 End of study reporting 28

14 STATISTICAL CONSIDERATIONS 28
14.1 Statistical Methods and Data Analysis 28
14.2 Analysis Data Sets 28
14.3 Patient demographics and baseline characteristics 29
14.4 Primary Objective 29
14.5 Interim Analysis 29
14.6 Sample Size Estimation 29

15 DOCUMENTATION 31
15.1 Case report forms and reports 31

16 ETHICAL CONSIDERATIONS 31
16.1 Informed consent and patient information 31
16.2 Premature withdrawal 32

17 ADMINISTRATIVE CONSIDERATIONS 32
17.1 Insurance 32
17.2 Monitoring 32
17.3 Auditing/inspecting 33
17.4 Archiving 33
17.5 Quality assurance 33
17.6 Trial activation procedure 34
17.7 Record retention 34
17.8 Drug Accountability 35

17.9 Samples banking 35
17.10 Trial registration 36
17.11 Modifications of the protocol 36
17.11.1 Scientific amendment 36
17.11.2 Safety amendment 36
17.11.3 Administrative amendment 36
17.12 Funding 36

18 PUBLICATION 37

19 CONFIDENTIALITY 37
19.1 Copyright 37
19.2 Confidentiality 38

20 REFERENCES 39

Appendix 1A Schedule of treatments 42
Group A, standard group 42
Group B, experimental group 43
Conditioning regimen: treatment schedule 44

Appendix 1B Supportive Care 45
VOD prophylaxis 45
VOD treatment (40) 45
GVHD prophylaxis 46
Prophylaxis of central nervous system adverse reactions 46
Prophylaxis of hemorrhagic cystitis 46
Antiemesis 47
Allogeneic SCT 47
Special management orders 47

Appendix 2 Karnofsky performance status(26) 48

Appendix 3 European Group for Blood and Marrow Transplantation (EBMT) risk score and Sorror comorbidity index 49
A) EBMT risk score (41) 49
B) Sorror comorbidity index (HSCT comorbidity index) (42) 51

Appendix 4 Veno-occlusive disease (VOD)(43) 53

Appendix 5 Graft versus Host Disease (GVHD)(27, 28) 54

Appendix 6 Assessment of the liver function 55

Appendix 7 Principal investigator's agreement 56

Appendix 8 Translational research analyses 58
Procedure for cytokines profiling 58
Handling and processing of samples 58
Plasma banking 58
Samples shipping 58
Procedure for genetic polymorphism screening/analysis 58
Handling and processing of samples 58
Samples shipping 59
DNA banking 59
Expression analysis of BER and GST pathway genes 59
Handling and processing of samples 59
Samples shipping 59
Procedure for alpha GST levels analysis and glutathione analysis 59
Handling and processing of samples 59
Samples shipping 59

Appendix 9 Checklist for collection of samples for pharmacogenomics and pharmacokinetics 60
Group A 60
Group B 61

Collection of blood samples 62
Plasma: GSTA0 / GSTA1 / GSTA2 62
DNA: DNA0 62
RNA: RNA0/RNA1/RNA2 62
Serum: Cyto0 / Cyto2 / Cyto3 / Cyto4 / Cyto5 63
Important notes 64

Appendix 10 Trial overview 65

4 TRIAL OVERVIEW (SYNOPSIS)

4.1 Objectives

The aim of this study is to test the hypothesis, that the order of application of Busulfan (BU) and Cyclophosphamide (CY) has an impact on toxicity after allogeneic Hematopoietic stem cell transplantation (HSCT) and that CY-BU reduces liver toxicity compared to BU-CY.

4.2 Primary endpoint

Liver toxicity at day 30, assessed as serum values of ASAT, ALAT, GGT, AP, and bilirubin.

4.3 Secondary endpoints

- Incidence and severity of Veno-occlusive Disease (VOD).
- Incidence and severity of acute Graft-versus-Host Disease (GVHD), by organ (skin, liver, gut) at day 30 and day 100
- Organ toxicity (definitions of the Common Terminology Criteria for Adverse Events (CTCAE), version 4.0) at day 30 and day 100
- Survival, relapse and non relapse mortality at day 30 and day 100

4.4 Trial design

Prospective randomized multicenter trial.

4.5 Selection of patients (most important criteria)

- Patients planned for an allogeneic HSCT with myeloablative conditioning
- Age 18 - 65 years
- Myeloid leukemia respectively related precursor neoplasms, or lymphoid neoplasms.
- HLA-identical sibling donor or matched unrelated (min. 10/10 Ag matched)

4.6 Trial duration

The inclusion of patients is planned to start in Q3 2012 and will stop after the inclusion of 36 patients in each arm, which is expected in Q2 2014.

4.7 Statistical considerations

The primary objective of the study is to estimate the difference in liver toxicity between the two study arms on day 30. The sample size estimation is based on the previous observational study and theoretical assumptions. Sample size was set to ensure at least 80 % power, $1 - \beta = 0.8$, at a

significance level = 0.05. For this study, 72 patients should be recruited to ensure 65 evaluable patients considering a drop-out rate of 9 %, as observed in the retrospective analysis.

4.8 Trial Treatment

Group A, standard group: conditioning regimen for allogeneic HSCT consists of intravenous BU 0.8 mg/kg administered every 6 hours as a 2-hours infusion (total 16 doses) on days -8 to -4 followed by intravenous CY 60 mg/kg as a 1-hour infusion on days -3 and -2.

Group B, experimental group: conditioning regimen for allogeneic HSCT consists of intravenous CY 60 mg/kg as a 1-hour infusion on days -8 and -7 followed by intravenous BU 0.8 mg/kg administered every 6 h as a 2-hours infusion ours (total 16 doses) on days -6 to -2.

5 SCHEME OF THE TRIAL

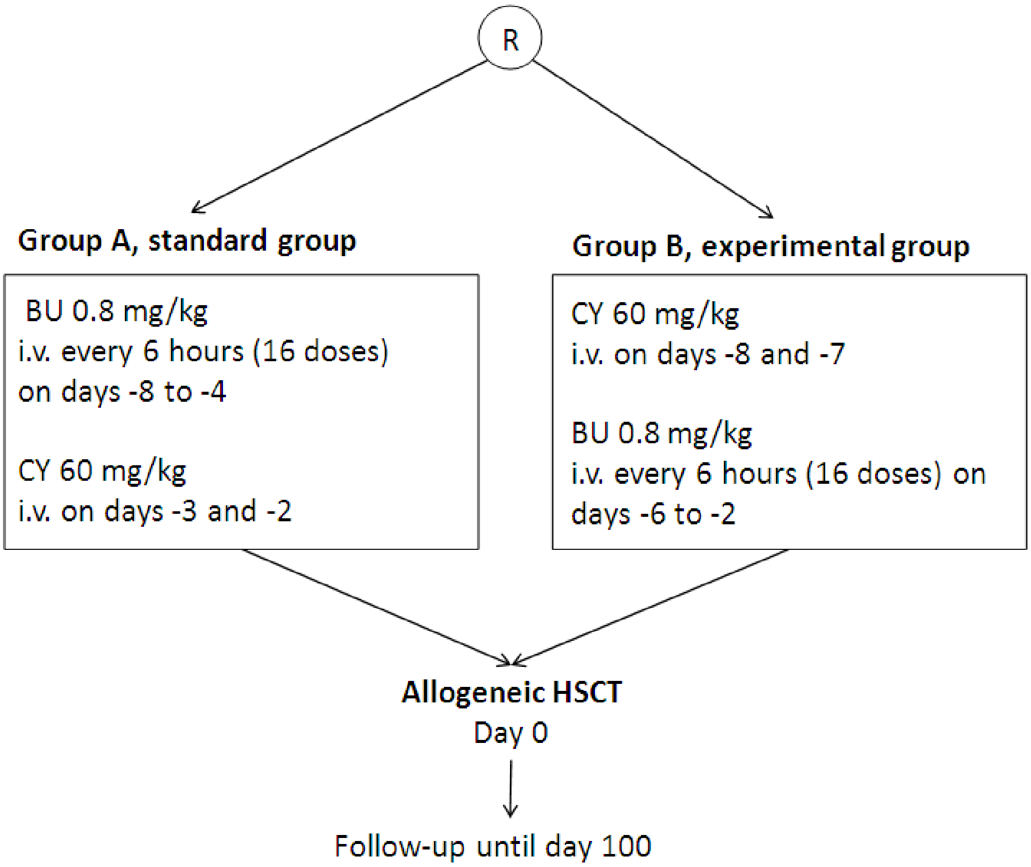

6 INTRODUCTION AND BACKGROUND

6.1 Therapy background

Busulfan (BU) - Cyclophosphamide (CY) is an established myeloablative-conditioning regimen for allogeneic hematopoietic stem cell transplantation (HSCT). It has a long-standing track record in the treatment of patients with leukemia and some congenital disorders and its advantages and disadvantages are well described. The antileukemic and immunosuppressive efficacy of BU-CY is considered to be equivalent to or even better than total body irradiation (TBI) in combination with CY.(1-3) Liver toxicity and hepatic veno-occlusive disease (VOD) are the most frequent early life-threatening complications associated with this approach.(4-6) Erratic absorption of oral BU has long been considered as a key factor for severe liver toxicity. Monitoring of serum levels has been advocated as a tool to reduce toxicity. Later on, the introduction of intravenous BU offered the advantage of easier administration with predictable pharmacokinetics and better tolerance than oral BU.(7) Both approaches reduced incidence and severity of liver complications but major problems remained.

6.2 Busulfan

6.2.1 Mechanism of action(8)

BU is an alkylating agent that interferes with DNA replication leading cells to apoptosis. BU enters the cells by passive diffusion. In vitro studies of cultured bone marrow cells demonstrate that BU at low concentrations is particularly cytotoxic for granulocytes and less cytotoxic to cells giving rise to other blood elements (platelets, lymphocytes, etc.). In aqueous media, BU hydrolyses to release the methyl-sulfonate groups at opposite ends of the four-carbon alkyl structure. This produces carbonium ions that alkylate DNA. Damage to DNA in rapidly dividing cells is considered to account for much of the cytotoxicity of BU. Busulfan reacts with guanine residues in DNA to form a four-carbon di-guanine DNA cross linkage which causes misreading of the DNA code and single-strand breakage. The degree of DNA cross-linkage and the cytotoxicity of the compound have been shown to be proportional to the dose. Replicating cells are most sensitive to BU in the G1 phase. Progression through the cell cycle is blocked in the G phase. By contrast, cultured cells in the S phase, which involves DNA synthesis, are not affected because the DNA repair system is active at that stage. Whilst resistant cells can eliminate the DNA cross-linkage produced by BU within 6 hours, in non-resistant cells similarly treated DNA cross-linkages remain unchanged even after 24 hours. The antileukemic and immunosuppressive efficacy of BU in combination with CY as conditioning regimen before allogeneic HSCT was confirmed in several studies.(1-3)

6.2.2 Absorption and bioavailability(8)

BU i.v. is administered as a two-hour infusion every 6 hours, mimicking the pharmacokinetic profile of the oral formulation where an average T_{max} at about 2 hours is usually observed. After 2-hour infusion of BU i.v. 0.8 mg/kg, peak concentrations at steady state ranged from 1.22 to 1.28 µg/ml, comparable to that observed after 1 mg/kg oral BU administration. The pharmacokinetic of BU i.v. and of the oral form were compared in a phase II study where oral BU (dose 1) versus BU i.v. (dose 9) have been administered in the same patient. Oral BU was characterized by erratic and variable absorption profiles whereas more consistent and predictable pharmacokinetic profiles were illustrated for the BU i.v. The variability (expressed as coefficient of variation) in T_{max} and C_{max} values were respectively 46% and 30% after oral administration whereas BU i.v. showed CV of 10% and 12%, respectively. As an intravenous formulation, i.v. Busulfan provides a 100% bioavailability. A comparison between oral and i.v. forms was investigated through a bioequivalence study using large PK datasets (120 i.v. patients versus 307 oral patients). The comparison between i.v. and oral route was conducted on both single and steady state doses. Equivalent systemic exposure of BU was demonstrated between 0.8 mg/kg i.v. and 1 mg/kg oral doses. This equivalence was established at dose 1 and maintained at steady state (see Figure 4). The calculated 90% confidence bioequivalence interval at steady state [0.96 to 1.07] was included in the required range [0.80 – 1.20]. Average bioavailability of the oral form (Myleran®) was calculated at about 80%.

6.2.3 Distribution(8)

During the clinical trials development, the mean volume of distribution (Vz) for BU i.v. ranged from 0.62 to 0.85 l/kg. These values are comparable to the volume of distribution reported for oral BU, which corresponds to about 66% of the body weight. BU achieves concentrations in the cerebrospinal fluid approximately equal to those in plasma. The drug also distributes freely into erythrocytes. Reversible plasma protein binding is low, 7.4% ± 4.9%, and irreversible binding to plasma elements, primarily albumin, has been estimated to be 32.4 ± 2.2%.

6.2.4 Metabolism(8)

BU i.v. is thought to be metabolised in a comparable way from that of the oral formulation. Therefore, information on metabolism can be obtained from oral BU investigations. BU metabolism has been studied most extensively in rats and the same metabolites were identified in human.

All metabolites arise from the initial conjugation of BU with glutathione in a glutathione S transferase (GST) catalysed reaction. The most active human GST catalysing this reaction is GSTA1. Three major metabolite peaks have been identified and none of them are thought to contribute significantly to

either efficacy or toxicity. The influence of GST polymorphism and specifically genetic variants of GSTA1, GSTM1, GSTP1 and GSTT1, on the PK of i.v. BU have been studied. These investigations have been performed in children. Literature data show no clear results; some authors reported a significant influence of GSTA1 polymorphism: a 30% decrease in BU i.v. clearance was calculated in carriers of GSTA1*B variant. Some authors also reported a lower BU i.v. clearance in GSTM1-null individuals. On the opposite and based on a larger sample size, other authors revealed that none of the polymorphism in the genes encoding of GSTA1, GSTM1, GSTP1 and GSTT1 had an influence on the BU elimination after i.v. administration.

6.2.5 Elimination(8)

Mean absolute clearance (CL) values for BU i.v. were consistent across studies, ranging from 2.25 ml/min/kg to 2.74 ml/min/kg. There was no significant difference in CL between dose 1 and dose 9, illustrating predictable and reproducible plasma exposure over the repeated administrations. Mass-balance elimination in human has been studied after both i.v. and oral BU administrations. It showed that whatever the route of administration is, BU dose is predominantly excreted in urine (essentially as metabolites) and negligible amounts of drug are recovered in faeces. Only 1 to 2% of an oral dose of BU are eliminated unchanged in human urine. Like oral BU, BU i.v. pharmacokinetic profile exhibits a mono-exponential decay after the end of infusion. The mean half-life of BU i.v. ranged from 2.83 to 3.90 hours. The steady-state concentration is achieved between the 3rd and the 4th administrations.

6.2.6 Drug-related adverse events(8)

Bone marrow depression, interstitial pneumonitis, nausea and emesis, diarrhoea, hepatic dysfunction, erythematous skin rash, allergic reaction, myastenia symptoms, cataract, infertility, alopecia, confusion, epileptic seizures (to be prevented by lorazepam prophylaxis, s. above), cardiomyopathy, atrophic bronchitis, adrenal hypofunction. Other rarely adverse events are reported in the Swissmedic reports for Busilvex®.(8)

The overall safety profile BU is mainly based on data from patients treated within clinical trials and on published literature or on NCI, EMEA or Swissmedic annual reports.

6.3 Cyclophosphamide

6.3.1 Metabolism and mechanism of action(9)

CY is biotransformed principally in the liver to active alkylating metabolites by a mixed function microsomal oxidase system. These metabolites interfere with the growth of susceptible rapidly proliferating malignant cells. The mechanism of action is thought to involve cross-linking of tumor

cell DNA. The antileukemic and immunosuppressive efficacy of CY in combination with BU as conditioning regimen before allogeneic HSCT was confirmed in several studies.(1-3)

6.3.2 Distribution(9)

Concentrations of CY metabolites reach a maximum in plasma 2 to 3 hours after an intravenous dose. CY achieves concentrations in the cerebrospinal fluid approximately equal to those in plasma. Plasma protein binding of unchanged drug is low (20%) but some metabolites are bound to an extent greater than 60%.

6.3.3 Elimination(9)

The mean half-life is 6-9 hours. CY is eliminated primarily in the form of metabolites, but from 5% to 25% of the dose is excreted in urine as unchanged drug. Several cytotoxic and non-cytotoxic metabolites have been identified in urine and in plasma.

6.3.4 Drug-related adverse events(9)

Bone marrow depression, fluid retention, cardiomyopathy, nausea and emesis, diarrhea, hepatic dysfunction, dizziness, blurred vision, hemorrhagic cystitis (prevented by forced diuresis and uromitexan, above), infertility, alopecia, erythematous skin rash. Other rarely adverse events are reported in the Swissmedic reports for Endoxan®.(9)

The overall safety profile CY is mainly based on data from patients treated within clinical trials and on published literature or on NCI, EMEA or Swissmedic annual reports.

6.4 Rationale for performing the trial

BU and CY are mainly metabolized in the liver. Interactions between these two drugs and with other commonly used drugs have been reported and are decisive for drug-related toxicity.(10-12) Clinical studies showed an increased liver toxicity, scored by the development of VOD and total serum bilirubin levels, non-relapse mortality and overall survival after hematopoietic cell transplantation in patients with increased exposure to toxic metabolites of CY.(13) It could also be shown that BU profoundly affects the metabolism of CY with higher levels of toxic metabolites of CY, if CY is given after BU.(7, 14) Pharmacological studies demonstrated that treatment with BU can decrease the levels of Glutathion-S Transferase (GST), a central player in the metabolism of toxic CY metabolites in hepatocytes.(15, 16) Furthermore, conditioning with BU-CY induced higher levels of liver enzymes and inflammatory cytokines.(17) This observation is important, because the proinflammatory cytokines, particularly IL-2 and TNF-alpha, may be one of the first steps in the development of acute graft-versus-host disease (GVHD).(18-20)

These theoretical considerations and pharmacological data indicate that application of BU may trigger liver toxicity of subsequent CY, and suggest that reverse order of CY-BU would be preferable. This was suggested several years ago in a mouse model (17) and in a non-randomized pediatric study of autologous HSCT where BU induced less liver toxicity when given as second drug.(11) Recent animal data confirmed this hypothesis (21), showing less liver toxicity and better outcomes when mice were treated with CY-BU compared to BU-CY. While CY-BU was not feasible in patients with oral BU in view of the high emetogenic potential of CY, it has become a possibility with the introduction of intravenous BU. We were therefore interested in exploring this concept and changed the order of drug application to CY-BU in 2006 in selected patients at the University Hospital of Basel. We recently analyzed in a retrospective study liver toxicity and outcome of these patients receiving BU-CY (before 2006) or CY-BU (after 2006), showing higher levels of liver function tests between day +10 and +30, as well as a higher cumulative incidence of VOD and transplant-related mortality in the BU-CY cohort.(22) These data support the concepts derived from animal models in favor of CY-BU compared to traditional BU-CY and form the basis for a prospective comparison.

In the present study, we want now to prospectively compare the order of application and liver toxicity in patients given BU and CY to confirm the aforementioned pharmacological, experimental and clinical results.

7 TRANSLATIONAL RESEARCH PROJECTS: BACKGROUND & METHODS

7.1 Cytokines profiling

Conditioning with BU and CY induced higher levels of inflammatory cytokines.(17) This observation is important, because the proinflammatory cytokines, particularly IL-2 and TNF-alpha, may be one of the first steps in the development of acute graft-versus-host disease (GVHD).(18-20) Cytokines profiling will be performed using Luminex® Assays (Luminex, Austin, USA) with a multiplex panel (Procarta® Immunoassay Kits - Plexable Human Cytokine, Chemokine and Growth Factors - Panel #1, Affymetrix Inc., Santa Clara, USA) to profile the expression of 54 key secreted inflammatory cytokines. This array includes IFNa, IFNb, IFNg, IL2, IL6, TNFa and TNFb.

7.2 Pharmakogenomics (Polymorphisms analysis)

Most of the drugs used to treat cancer are metabolised by hepatic enzymes such as cytochrome P450 (CYP450) or Glutathione-S-Transferase (GST). These enzymatic pathways can be more or less active in

the drug's metabolism according to the given polymorphism of each patient (pharmacogenomics) and their activity could be altered by drug-drug interactions.

Busulfan and cyclophosphamide are alkylating agents acting by cross linking the guanine bases in double stranded DNA and damage induced by these agents is commonly repaired by the base excision repair (BER) pathway.(23) These agents are less effective on cells in S phase, because of the activity of DNA repair enzymes. There is a wide list of enzymes involved in DNA repair acting via different pathways such as BER. If these enzymes show altered activity due to variations in their genes, then the cells may become sensitive/resistant to these alkylating agents. The genetic variations in these genes may also have an impact on the treatment outcome related to alkylating agents.

The current hypothesis is that some functional polymorphisms of genes (GST/CYPs), which control important enzymes in BU/CY metabolism, contribute to the observed interindividual variability in pharmacokinetics of these drugs. This variability can hence predict the resistance as well as the toxicity from a drug in patients who have cancer. The pharmacokinetic profile of different drugs, which have a hepatic metabolism, can be dramatically modified by these polymorphisms. In addition to these the variations and /or expression of the genes coding for the proteins involved in BER pathway might have an influence on the efficacy of BU and CY.

Hence, the current study is aimed at investigating the genetic variants and/or expression of the GST/CYP450 and the base excision repair (BER) pathway genes for their potential as molecular markers for predicting the efficacy and outcome of myeloablative therapy with BU and CY. We would like to study as well the relationship between the pharmacokinetics and pharmacogenetics of busulfan and cyclophosphamide.

7.3 alpha GST levels and glutathione analysis

Plasma alpha GST will be measured using HEPKIT alpha (a quantitative enzyme immunoassay from Biotrin International, Dublin, Ireland). The test procedure is based on the sequential addition of sample, anti alpha GST enzyme conjugate and substrate to microassay wells coated with anti alpha GST. The resultant color intensity is proportional to the amount of alpha GST present in the sample. Assay range is 0-40 microg/L, equivalent to 0-200 microg/L in samples diluted 1:5. HEPKIT alpha is highly specific and no cross reactivity has been observed with either mu or pi isoforms of GST.(24) GST is stable in serum and can be stored 15 months at -20°C. Glutathione will be measured using a spectrophometric/ colorimetric method.

7.4 Expression analysis of BER and GST pathway genes and their influence on outcome to treatment

The RNA extraction will be performed for all the samples at 9-12 months of storage point using Trizol reagent and will be converted to cDNA using high capacitance cDNA Archive kit (Applied Biosystems, Foster City, USA) and will be stored at -80°C till further anlaysis.

Gene expression analysis will be performed using SYBR green chemistry on a SteponePlus real time PCR system (Applied Biosystems, Foster City, USA) with gene specific primers designed for the genes involved in BER pathway, GST and other genes of DNA repair pathways to be playing a role in repair of DNA damage produced by BU and CY.

8 OBJECTIVES AND ENDPOINTS

8.1 Study Aim

The primary study goal of this prospective randomized study is to test the hypothesis, that the order of application of BU and CY has an impact on toxicity after allogeneic HSCT and that CY-BU reduces liver toxicity compared to BU-CY. Reduced liver toxicity is considered as a prerequisite for reduced liver GVHD and improved survival.

8.2 Primary endpoint

Liver toxicity at day 30, assessed as absolute serum values of ASAT, ALAT, GGT, AP, bilirubin at day 30.

8.3 Secondary endpoints

i) Maximum serum values of ASAT, ALAT, GGT, AP, bilirubin at any time between day 0 and day 30

ii) Cumulative serum values of ASAT, ALAT, GGT, AP, bilirubin for days 0, 10, 20 and 30

iii) Incidence and severity of VOD at day 30

iv) Incidence and severity of acute GVHD, by organ (skin, liver, gut) at day 30 and day 100

v) Organ toxicity at day 30 and day 100

vi) Survival, relapse and non-relapse mortality at day 30 and day 100

8.4 Additional research questions

8.4.1 Cytokines measurement

In animal models could be shown that the conditioning regimen with BU-CY induced higher levels of liver enzymes and inflammatory cytokines in comparison to a conditioning regimen with CY-BU.(17) This observation is important, because the proinflammatory cytokines, particularly IL-2 and TNF-alpha, may be one of the first steps in the development of acute graft-versus-host disease (GVHD).(18-20)

To test this correlation between order of application of the conditioning regimen and the levels of proinflammatory cytokines as well as the correlation between levels of cytokines and development of acute GVHD, plasma samples will be collected at different time points. At the end of the study the plasma samples will be analyzed with an ELISA test for proinflammatory cytokines as described previously.

8.4.2 Pharmacogenomics (polymorphisms)

Most of the drugs used to treat cancer are metabolised by hepatic enzymes such as cytochrome P450 (CYP450) or GST. These enzymatic pathways can be more or less active in the drug's metabolism according to the given polymorphism of each patient (pharmacogenomics). The current hypothesis is that some functional polymorphisms of genes, which control important enzymes in BU and CY metabolism, contribute to the observed interindividual variability in toxicity after allogeneic HSCT. The pharmacokinetic profile of different drugs, which have a hepatic metabolism, can be dramatically modified by these polymorphisms.

In order to gain further insight into the role of these polymorphisms and their ability to predict the clinical response parameters (graft rejection, relapse following bone marrow transplantation, liver toxicity, VOD) we propose to correlate all these values with GST polymorphisms.

9 TRIAL DESIGN, DURATION AND TERMINATION

The study is designed as a prospective randomized multicenter study comparing CY-BU (group B, experimental group) as conditioning before allogeneic HSCT for myeloid leukemia with BU-CY (group A, standard group).

The inclusion of patients is planned to start in Q3 2012 and will stop after the inclusion of 36 patients in each arm (see statistical considerations), which is expected in Q2 2014. End of trial (last patient, last visit) is expected for Q2 2015. All patients will be followed up for 100 days after allogeneic HSCT.

10 SELECTION OF PATIENTS

10.1 Patient population

Adult patients receiving an allogeneic stem cell transplantation for myeloid leukemia at the Division of Hematology at the Department of Internal Medicine at the University Hospital Basel (UHBS), Division of Hematology at the Department of Internal Medicine at the University Hospital Geneva (HUG), and Division of Hematology at the Department of Internal Medicine at the University Hospital Zürich (USZ).

10.2 Inclusion criteria

- Patients planned to undergo an allogeneic HSCT with myeloablative conditioning
- Age 18 - 65 years
- Myeloid leukemia respectively related precursor neoplasms (acute myeloid leukemia, chronic myeloid leukemia, myelodysplastic syndrome), or lymphoid neoplasms (acute lymphoblastic leukemia/lymphoma, mature B-/T-/NK-cell neoplasms).
- HLA-identical sibling donor or matched unrelated (min. 10/10 Ag matched)
- Patients with a history of hepatitis might be included, if no contraindication for HSCT exists.
- Patient must give written informed consent

10.3 Exclusion criteria

- Indication other than myeloid leukemia respectively related precursor neoplasms, or lymphoid neoplasms.
- Severe liver damage for > 2 weeks (bilirubin > 3xULN or AST/ALT > 5xULN)
- HIV infection
- Donor other than HLA-identical sibling or min. 10/10 matched unrelated donor
- Pregnant or lactating women
- Lack of written informed consent

10.4 Enrollment

Prior to enrollment, the following steps have to be performed:

- Check the eligibility criteria
- Obtain written informed consent
- Fill in the patient screening, enrollment and identification list

Enrolment of study patients will be documented in the electronic data capture (EDC) system secuTrial®. Study personnel will be provided with an individual login and password to access the electronic data capture system

10.5 After enrollment

Allogeneic HSCT should be performed within 3 months from subject registration.

10.6 Randomization

Patients will be randomized (1:1) to receive BU-CY (group A, standard group) or CY-BU (group B, experimental group) as conditioning regimen for allogeneic HSCT.

Randomization will be performed online via the electronic data capture system (secuTrial®).

10.7 Stratification

Patients will be stratified for randomization by center and donor type (related vs. unrelated).

11 TRIAL TREATMENT

11.1 Therapy schedules

The BU-CY conditioning (standard group A) regimen consists of intravenous BU 0.8 mg/kg administered every 6 hours (total 16 doses) on days -8 to -4 followed by intravenous CY 60 mg/kg on days -3 and -2.

The CY-BU conditioning regimen (experimental group B) consisted of intravenous CY 60 mg/kg on days -8 and -7 followed by intravenous BU 0.8 mg/kg administered every 6 hours (total 16 doses) on days -6 to -2.

Conditioning regimen group A (s. Appendix 1)

Agent	Dose	Route	Day(s)
Busilvex®	0.8 mg/kg every 6 h	2 h infusion i.v.	-8 thru -4
Endoxan®	60 mg/kg/day	1 h infusion i.v.	-3 thru -2
Allogeneic HSCT			0

Busilvex® (BU): to be dissolved in 0.9% NaCl to a final concentration of 0.5 mg/ml

Endoxan® (CY): to be dissolved in 500 ml 5% glucose or 0.9% NaCl

A 24 hours delay should be implemented between the last dose of BU and the first dose of CY

Conditioning regimen group B (s. Appendix 1).

Agent	Dose	Route	Day(s)
Endoxan®	60 mg/kg/day	1 h infusion i.v.	-8 thru -7
Busilvex®	0.8 mg/kg every 6 h	2 h infusion i.v.	-6 thru -2
Allogeneic HSCT			0

Endoxan® (CY): to be dissolved in 500 ml 5% glucose or 0.9% NaCl

Busilvex® (BU): to be dissolved in 0.9% NaCl to a final concentration of 0.5 mg/ml

A 24 hours delay should be implemented between the last dose of CY and first dose of BU.

11.2 Handling, preparation and administration of CY and BU

For handling, preparation and administration refer to the products information.(8, 9) SUVA guidelines on handling of cytostatics have to be followed.(25)

CY have to be dissolved in 500 ml 5% glucose or 0.9% NaCl; BU have to be diluted in NaCl 0.9% to a final concentration of 0.5mg/ml for stability reasons, as described in the products information of Busilvex®.(8) A 24 hours delay should be implemented between the BU and CY resp. CY and BU to decrease the eventuality of interactions between the two drugs and to increase the safety.

11.3 Allogeneic HSCT

The allogeneic HSCT will be performed at day 0 per intravenous route in approximately 15-30 min according to the institutional guidelines of the different centers. The graft will be checked for cell count.

11.4 Supportive care

Supportive care will be according to standard procedures described in appendix 1B.

12 EVALUATIONS AND INVESTIGATIONS BEFORE, DURING AND AFTER TRIAL

12.1 Pretreatment evaluations and procedures

Informed consent must be obtained before the first examination.

12.2 Evaluations to be performed within 21 days before treatment

Study subjects will be screened for eligibility before registration in database.

- Locally documented leukemia diagnosis, incl. disease stage
- Assessment of liver function by measuring levels of bilirubin and liver enzymes (AST, ALT, GGT, and AP).
- Serology for HIV, hepatitis A, B and C, CMV, EBV

12.3 Evaluations prior to start of the conditioning regimen

- Medical history, including previous chemotherapy or radiotherapy, antecedent hematological or oncological disease, hepatic diseases, prior drugs
- Physical examination including body weight, height, hepatosplenomegaly, Karnofsky performance status(26) (s. Appendix 2)
- Document EBMT and Sorror risk scores (s. Appendix 3)
- Assessment of liver function by measuring levels of bilirubin and liver enzymes (AST, ALT, GGT, and AP) as well as ferritin, albumin and coagulation studies including protrombin time (PT), partial thromboplastin time (PTT), and fibrinogen.
- Blood samples for Cytokines profiling (s. Appendix 8 and 9)
- Blood samples for Pharmakogenomics (s. Appendix 8 and 9)
- Pregnancy test for women
- Imaging of the liver (ultrasound or CT-scan)

- Other examinations are performed according to the institutional guidelines of the different centers.

12.4 Evaluations during trial

- Physical examination at day 0, 30 and 100 including Karnofsky performance status(26) (s. Appendix 2)
- Assessment of liver function by measuring levels of bilirubin, liver enzymes (AST, ALT, GGT, and AP), ferritin and albumin at least at day 0, 10, 20 and 30.
- Blood samples for Cytokines profiling (s. Appendix 8 and 9)
- Blood samples for Pharmakogenomics (s. Appendix 8 and 9)
- Blood analyses for the pharmacokinetics of BU will be routinely performed before and after the application of the third dose of BU according to the institutional guidelines of the center. The adjustment of BU dosis due to the BU pharmacokinetics will be performed according to the institutional guidelines of the center. Further blood analyses for the pharmacokinetics of BU are dependent on the first pharmacokinetic analysis and are performed independently of this study and according to the institutional guidelines of the center.
- Documentation of the presence of the three clinical manifestations of VOD according to the Seattle criteria (s. Appendix 4)
- Documentation on adverse events observed.
- Severity and organ inolvement of acute GVHD will be assessed daily during the hospitalization and weekly for out-patients according to established criteria (27, 28) (s. Appendix 5). Whenever possible, diagnosis of GVHD should be confirmed by skin or gut biopsy.

12.5 Evaluations after *treatment*

- Follow-up information for survival status, further treatments and, if applicable, progression/relapse at day 30 and 100. All responses, changes in response status and progression/relapse have to be documented on the eCRF.
- Other examinations are performed according to the institutional guidelines of the different centers.

12.6 Sampling for pharmacogenomics, and cytokines profiling

Sampling and processing details are specified in Appendix 8.

13 SAFETY

13.1 Adverse events: Definitions

13.1.1 Adverse Event (AE):

Any untoward medical occurrence in a clinical trial subject administered a study drug and which does not necessarily have a causal relationship with this treatment. An AE can therefore be any unfavourable and unintended sign (including an abnormal laboratory finding), symptom, or disease temporally associated with the trial treatment, whether or not considered related to the this treatment. .

13.1.2 Adverse Reaction (AR):

All untoward and unintended responses to a study drug judged by sponsor-investigator as having a reasonable causal relationship to the study drug. The expression reasonable causal relationship means to convey in general that there is evidence or argument to suggest a causal relationship.

13.1.3 Serious Adverse Event (SAE) or Serious Adverse Drug Reaction (SADR):

A SAE includes any of the events listed in the table below and occurring during trial and up to 30 days after the last follow-up visit:

- All deaths
- Life-threatening (the patient was at immediate risk of death from the event as it occurred. It does not include an event that, had it occurred in a more serious form, might have caused death).
- Requires inpatient hospitalization for ≥ 24 h (Events not considered to be serious adverse events are hospitalizations occurring under the following circumstances: elective surgery or part of the normal treatment or monitoring of the trial treatment or progressive disease)
- Prolongation of an existing hospitalization
- Disabling Includes persistent or relevant disability or incapacity
- Second primary cancer (Any new malignancy other than a relapse of the current tumor)
- Other medically significant condition (Important adverse events that are not immediately life-threatening or do not result in death or hospitalization but may jeopardize the patient or may require intervention to prevent one of the other outcomes listed above).

SADR are all SAEs considered to be related (possibly, probably, definitively) to the trial treatment.

13.1.4 Suspected Unexpected Serious Adverse Reaction (SUSAR):

SUSARs are serious adverse reactions that are assessed as unexpected on the basis of the applicable Swiss product information,(8, 9) and the European summary of product characteristics.(29)

13.2 Causality assessment of adverse events

Most adverse events and adverse reactions that occur in the study, whether they are serious or not, will be expected treatment-related toxicities due to the drugs used in this study. The assignment of the causality should be made by the investigator responsible for the care of the participant using the definitions in the table below.

Unrelated:	The AE is clearly not related to the trial treatment. The AE is completely independent of trial treatment and/or evidence exists that the event is definitely related to another etiology.
Unlikely	The AE is doubtfully related to the trial treatment. Temporal association between the AE and the trial treatment and the nature of the event is such that the trial treatment is not likely to have had any reasonable association with the observed illness/event (cause and effect relationship improbable but not impossible).
Possible	The AE may be related to the trial treatment. Less clear temporal association; other etiologies also possible.
Probable	The AE is likely related to the trial treatment. Clear-cut temporal association and a potential alternative etiology is not apparent.
Definitely	The AE is clearly related to the trial treatment. Clear-cut temporal association and no other possible cause.
Not assessable	There is insufficient or incomplete evidence to make a clinical judgment of the causal relationship.

13.3 Handling of adverse events

Subjects with AEs will be treated appropriately. Abnormal laboratory values will be repeated until normal or until the abnormality can be explained and the subject’s safety is not at risk. In cases of a medical emergency, treatment is available in house and includes the 24h-availability of the reanimation team and intensive care facilities. In case of a fatal SAE, investigator will provide Clinical

Trial Unit (CTU) Basel all requested information, who will forward this information to the independent ethics committee within the legally fixed timeframes.

13.3.1 Medical follow-up of adverse events

The investigator will ensure that the subject receives medical follow-up as necessary until the condition has stabilized or returned to normal state, even if the period of the trial is over.

13.3.2 Data Safety Monitoring Committee (DSMC)

To ensure the safety of the participating study subjects a DSMC will review on the basis of the FDA's Guidance for Clinical Trial Sponsors(30) accumulating safety data (SAEs) from the trial. The DSMC advises the sponsor regarding the continuing safety of trial. Concerns about the extent and type of adverse events observed may lead to early termination of the trial when the DSMC judges that the potential benefits of the intervention are unlikely to outweigh the risks. Otherwise, the DSMC may recommend measures that might reduce the risk of adverse events.

The DSMC is composed of clinicians with expertise in the field of allogeneic HSCT and one biostatistician. All the members of the DSMC have no potential conflicts of interest that could impede objectivity in the evaluation of the safety data of the trial.

The members of the DSMC are:

- Prof. Dr. med. Mario Bargetzi, Head (Chefarzt) of Hematology Division, University Clinic for Medicine, Kantonsspital Aarau, 5001 Aarau, Switzerland and former chairman of the Ethic Committee Aargau / Solothurn (Prof. Dr. med. M. Bargetzi will be the appointed DSMC chairman)
- PD Dr. med. Tayfun Güngör, Consultant Physician (Leitender Arzt), Division Bone Marrow Transplantation, University Children's Hospital Zürich, 8032 Zürich, Switzerland
- Dr. med. Thomas Lehmann, Consultant Physician (Leitender Arzt), Hematology Division, Kantonsspital St. Gallen, 9007 St. Gallen, Switzerland
- PD Dr. med. Philipp Schütz, Epidemiologist, Master of Public Health, Endocrinology Division, University Clinic for Medicine, Kantonsspital Aarau, 5001 Aarau, SwitzerlandCoding and Reporting to local and national authorities

13.3.3 SAE coding

SAEs are coded with the NCI Common Terminology Criteria for Adverse Events (CTCAE) version 4.0, and assigned a grade (from 1 = mild to 5 = death related to AE) as well as a relationship to trial treatment. The NCI CTCAE version 4.0 (as pdf) as well as instructions on how to use the criteria can be found on: *http://ctep.cancer.gov/protocolDevelopment/electronic_applications/ctc.htm*

13.3.4 SAE and SUSAR reporting

In case of an SAE, relationship to the study drug will be assessed by the investigator using the definitions mentioned above. The principle investigator (PI) ensures complete collection and documentation information concerning the SAE on a standard SAE form. Completes information will then be forwarded to CTU Basel who reports to the independent ethic committee and Swissmedic on behalf of the PI according to guidelines within 7 or 15 days.

13.3.5 Annual safety report

An annual safety report will be provided to the competent authority and to the independent ethics committee by CTU Basel.

13.3.6 End of study reporting

The PI will inform the independent ethics committee and Swissmedic when the study ends within 90 days or 15 days if the study is terminated early. A final report is sent to Swissmedic within six months of study termination by the investigator.

14 STATISTICAL CONSIDERATIONS

14.1 Statistical Methods and Data Analysis

Detailed methodology for summaries and statistical analyses of the data collected in this study will be documented in a statistical analysis plan. The analysis plan will be finalized before database closure and will be under version control at the Clinical Trial Unit, University Hospital Basel.

14.2 Analysis Data Sets

The all-subjects-randomized (ASR) set will be used as a safety set. The intention to treat (ITT) set consists of patients in the ASR without any major protocol deviation. Treatment will be assigned as indicated in the randomization list. If there is for whatever reason a difference between the applied procedure and the randomization list, a per protocol (PP) set will be defined for all patients in the ASR without any major protocol deviation. Treatment will be assigned according to the procedure the patient received. Detailed justification and data listings will be provided for patients who were

not randomized although they had signed the consent form. For secondary objectives further analysis sets may be defined.

14.3 Patient demographics and baseline characteristics

Demographics and relevant baseline variables will be presented for the ASR set. Categorical data will be presented as frequencies and percentages. For continuous variables, the lower and upper quartile as well as the median will be presented.

14.4 Primary Objective

The primary objective of the study is to estimate the difference in ALT concentration (primary endpoint) between the two study arms on day 30. The difference in location will be estimated together with a confidence interval(31) and will be tested with a Wilcoxon Test. The analysis will be performed on the ITT set, which is restricted to patients with follow-up measurements of the ALT-concentration on day 20 or 30, or both. For patients with missing ALT-concentration on day 30, the missing measurement will be replaced by one before day 30.

Supportive analysis I: In addition, the difference in ALT-concentration together with a 95% confidence interval will be estimated using a linear regression model with the baseline value of the ALT-concentration as covariate.

Supportive analysis II: If a PP set was defined all analyses will be repeated on the PP set.

14.5 Interim Analysis

No interim analysis is planned.

14.6 Sample Size Estimation

Sample size was estimated to be able to test if there is a significant difference in ALT concentration between the experimental group CY-BU and the standard group BU-CY. Sample size calculation was based on data of 75 patients used for a retrospective analysis by Cantoni et al.(22), with a very similar objective as the planned prospective study. In the retrospective study, a reduction of the ALT concentration in the CY-BU group of 47.14 % was observed. Sample size was calculated with a semi-parametric resampling method as suggested by Davison & Hinkley.(32) This allows at the same time to account non-parametrically for the pilot data set and parametrically for the treatment shift, θ. Each sample size,

$n_{i=1,....,21}$ = 40, …., 120, was evaluated by sampling 3333 times n_i individual patients with replacement from the data after centering the data on the ALT-concentration in the BU-CY group. Half of the

patients were randomly assigned to each group and the effect size, θ = -35, was added to the patients in the CY-BU group. Thereafter a Wilcoxon test was used to test for a difference between the two groups. Sample size was set to ensure at least 80 % power, 1 – β = 0.8, at a significance level = 0.05. For this study, 72 patients should be recruited to ensure 65 evaluable patients considering a drop-out rate of 9 %, as observed in a retrospective analysis. Figure 1 presents how sensitive the sample size is with respect to the expected reduction of the ALT concentration.

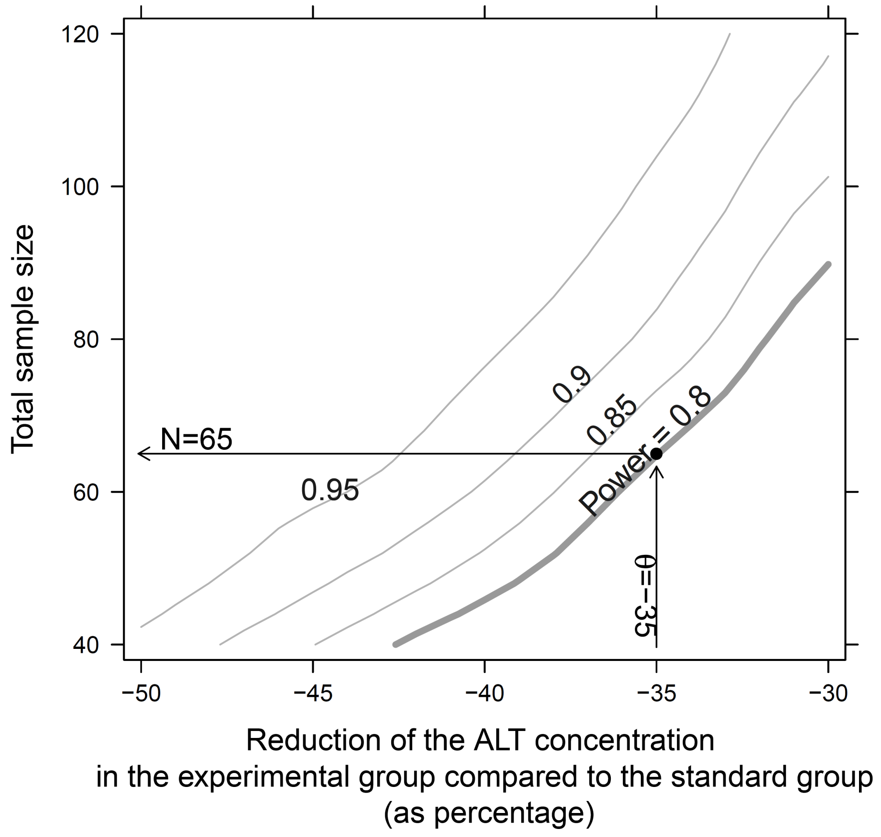

Figure 1 - Sensitivity of the sample size with respect to the reduction of the ALT concentration in the experimental group compared to the standard group. Numbers on the curve denote the corresponding power. Arrows indicate how to read the figure. The curve is smoothed and for illustrative purpose only.

15 DOCUMENTATION

15.1 Case report forms and reports

The reports are based on the EBMT MED-A and MED-B forms; in addition electronic case report forms (eCRF) specifically created for this trial are used for items not mentioned in the EBMT MED-A and MED-B forms. The monitor will collect a copy of the EBMT MED-A and MED-B forms.

Centers must use a patient screening, enrollment and identification list in order to allow identification of a patient and proper usage of initials. This list must be kept at the center in the investigator site file.

16 ETHICAL CONSIDERATIONS

This protocol was written, and the trial is to be performed in accordance with the Declaration of Helsinki, the Guidelines of Good Clinical Practice issued by ICH and Swiss regulatory authorities requirements.(33-36) Before planning to enter any patients into this trial, the investigator has to make sure that the trial has been approved by the local ethics committee and that their center has officially been opened by Swissmedic, if applicable. The local investigator is responsible for ensuring that the study will be conducted in accordance with the protocol, the ethical principles of the Declaration of Helsinki,(35) current ICH guidelines on Good Clinical Practice (GCP),(36) and applicable regulatory requirements.

There should be no ethical conflicts. Patients will be either treated with the standard approach (group A) or with the novel approach (group B). There are some indications that the novel approach will be less toxic but there is no proof so far.

16.1 Informed consent and patient information

The informed consent procedure must conform to the guidelines on Good Clinical Practice issued by ICH(36) and Swissmedic. All patients will be informed of the aims and procedures of the trial, the possible adverse events, how to react in case an adverse event occurs, and possible hazards to which he/she will be exposed. They will be informed as to the strict confidentiality of their patient data, but they need to know that their medical records may be reviewed for trial purposes by authorized individuals other than their treating physician. An investigator must provide the patient with sufficient opportunity to consider whether or not to participate and minimize the possibility of coercion or undue influence. The information provided shall be in a language intelligible to the patient and may not include any content that appears to waive any of the patient's legal rights, or appears to release the investigator, the sponsor, or the institution from liability for negligence. It will

be emphasized that participation is voluntary and that the patient is allowed to refuse further participation in the trial whenever he/she wants. This will not prejudice the patient's subsequent care. Informed consent shall be obtained on a written form approved by the local ethics committee and signed by the patient. The patient information as well as a copy of the signed and dated informed consent will be handed to the patient.

16.2 Premature withdrawal

Patients have the right to refuse further treatment for any reason and at any time. Patients who decide to withdraw from the trial should be asked whether they also want to withdraw their consent for their data to be used for the follow-up assessments. For the patient’s security, a last examination should be performed.

Patients may be withdrawn at any time from trial treatment at the discretion of the investigator due to a serious adverse event, or based on any other relevant medical condition.

17 ADMINISTRATIVE CONSIDERATIONS

17.1 Insurance

The Sponsor will indemnify patients for damages they have suffered as participants in the trial. For this purpose, the Sponsor has taken out a special insurance for clinical trials.

17.2 Monitoring

The monitor will contact and visit the centers regularly. He/she will be allowed to inspect the various records of the trial in accordance with local requirements. All source documents must be accessible for monitoring. The monitor will maintain patient confidentiality. For this trial the expected average monitoring visit frequency is at least every 3 months during the treatment phase. This frequency may be adjusted based on the recruitment and the stage of the trial.

Before enrollment of the first patient, a trial initiation visit will take place. The objective of this visit is to meet the local staff involved in the conduct of the trial (including sub-investigators, research nurse, clinical research coordinator, pharmacist), to describe the main features of the protocol, the use of the case report forms, the practicalities of the trial and to distribute the trial-specific investigator site file (ISF). The initiation visit has to be documented by the monitor on the ‘checklist for initiation visit’.

During monitoring visits, 100% source data verification (SDV) will be performed for the first patient at a center. If no major discrepancies are found, SDV may be reduced and only the following data will be verified for every patient: Informed consent, Inclusion/exclusion criteria, Serious Adverse Events (SAEs), Serious Adverse Drug Reactions (SADRs), Primary endpoint .

In case of inadequate data quality, 100% SDV will be performed for further patients until acceptable data quality is again obtained. The monitors must provide all monitoring reports to the Trial Chairperson within 2 weeks from the visit.

At the end of the trial the monitor will make a study closing visit to all sites to ensure that all documentation is complete.

Details regarding the monitoring activities will be specified in the separate document Monitoring Plan.

17.3 Auditing/inspecting

Authorities have the right to perform inspections, and the Coordinating centers as well as the independent ethics committees have the right to perform on-site auditing during working hours upon reasonable prior notice. Source data must be accessible for inspection and auditing visits.

17.4 Archiving

The investigator is responsible for archiving of the Investigator's file (including the original signed informed consent forms of all participants) for at least 10 years after the end or the termination of the trial.

17.5 Quality assurance

Several procedures guarantee quality of trial conduct:

- Reviews of protocol and forms according to standard operating procedures
- Requirements for principal investigators for participation: signed and dated CV and trial-specific agreement
- Validation of database and statistical analysis
- Data will be entered in an electronic CRF. Computerized and manual consistency checks will be performed; in case of inconsistencies queries will be issued.
- Data review by the trial chair or a delegated person (all CRF will be reviewed and checked on medical content)
- Safety monitoring

- An authorization list must be kept at the center
- The trial will be monitored (SDV, verification of informed consent etc.) by personnel designated
- The involved laboratories operate in compliance with the Good Laboratory Practice (GLP) principles,(37) and perform analysis by routine assays in accordance with the procedures of the International Federation of Clinical Chemistry (IFCC).(38)

17.6 Trial activation procedure

Prior to activation, centers have to submit the following documents to the CTU:

- The signed and dated trial-specific Principal Investigator's Agreement (see Appendix 7), indicating that they will fully comply with the protocol, including an estimation of their annual accrual and additional items
- Signed and dated CV of the principal investigator
- A copy of the certification of GCP training course
- A copy of the "Basisformular zur Einreichung eines biomedizinischen Forschungsprojektes/Formulaire de base pour la soumission d'un projet de recherche biomédicale" that has been submitted to the local ethics committee.
- Ethics approval for the site, i.e. a positive statement on the "Formular für die Beschlussmitteilung der Ethikkommission/Formulaire d'avis de la Commission d'éthique de la recherche"
- Approved patient information and informed consent, as well as all previous versions (if any)
- Copies of all documents submitted to the ethics committee. Please refer to the "Basisformular zur Einreichung eines biomedizinischen Forschungsprojektes".
- Once all these documents of a site are submitted to the CTU, they will be forwarded to Swissmedic, if applicable.
- The investigator will only be allowed to register patients into the trial after Swissmedic has approved the center (if applicable).

17.7 Record retention

The center will retain copies of the patient trial records (eCRF, MED-A and MED-B form patient informed consent statement, laboratory printouts, drug inventory logs, and all other information collected during the trial) and documentation until at least 10 years after the termination of the trial. In the event that the investigator retires or changes employment, custody of the records may be transferred to another competent person who will accept responsibility for those records. Written

notice of such transfer will be given to the CTU and the ethics committee. The CTU will notify the regulatory authorities.

17.8 Drug Accountability

Drug supplies, which will be provided by the hospital pharmacy, University Hospital Basel, will be kept in a secure, limited access storage area under the storage conditions appropriate for the study drug. The research site staff will maintain records of the product's delivery to the trial site, the inventory at the site, the use by each subject, and the return to the hospital pharmacy or alternative disposition of unused product(s). These records will include dates, quantities, batch/serial numbers, and expiry dates. The research site staff will maintain records that document adequately that the subjects were provided the doses specified by the study protocol and reconcile all investigational product(s) received from the hospital pharmacy. At the time of return to the hospital pharmacy, the research site staff must verify that no remaining supplies are in the investigator's possession.

17.9 Samples banking

Samples will be banked and kept for 20 years for future analyses in connection with polymporphisms or cytokines and conditioning regimen after allogeneic HSCT according to the SAMW guidelines.(39) The biobanking is described in detail in the "Biobankreglement" and the corresponding patient informed consent form. The plasma banking for cytokines profiling will be done in the University Hospital of Basel, Switzerland. The DNA Banking will be done in HUG Geneva University Hospital, Geneva, Switzerland.

The patient retains the right to have the sample material destroyed at any time by contacting the local investigator. However, already obtained data from this material can be used for intended analyses. The sponsor will be the exclusive owner of any data, discoveries, or derivative materials from the sample materials and is responsible for the destruction of the samples at the request of the research patient through the local investigator or at the end of the storage period. The local investigator will provide the principal investigator/ sponsor with the required trial and patient numbers (UPN) so that any remaining blood and any other components from the cells can be located and destroyed.

If a commercial product is developed from this research project, the sponsor will own the commercial product. The patient will have no commercial rights to such product and will have no

commercial rights to the data, information, discoveries, or derivative materials gained or produced from the sample.

Any new analysis on these samples not planned in this protocol has to be approved by the steering committee,by the relevant ethics committees and by Swissmedic, if applicable.

17.10 Trial registration

The trial is registered in the NIH's ClinicalTrials.gov registry (NCT01779882).

17.11 Modifications of the protocol

17.11.1 Scientific amendment

Any amendment which may have an impact on the conduct of the trial, the potential benefit of the trial, or may affect patient safety, including changes of trial objectives, trial design, patient population, sample sizes, trial procedures, or significant administrative aspects must have been accepted by the SBST Board. Such an amendment is termed scientific amendment and must have the approval of the respective ethics committee and Swissmedic prior to implementation.

17.11.2 Safety amendment

A safety amendment is a special kind of scientific amendment, which is released when it is necessary to eliminate immediate hazards to trial participants. A safety amendment requires immediate implementation at local sites, before approval of local ethics committee and Swissmedic has been given.

17.11.3 Administrative amendment

Amendments including administrative changes such as minor corrections and/or clarifications that have no effect on the way the trial is to be conducted have to be submitted to the ethics committee. Such an amendment is called administrative amendment and may be implemented with immediate effect. A letter of receipt of the ethics committee has to be forwarded to the CTU.

17.12 Funding

Baxter SA and Robapharm/Pierre Fabre SA support the study with unrestricted funding for the administrative costs.

18 PUBLICATION

The trial results will always be submitted for publication in a peer reviewed scientific journal regardless of the outcome of the trial – unless the trial was terminated prematurely and did not yield sufficient data for a publication. The final publication of the trial results will be written by the Trial Chairperson, the Co-Chairperson, the Principal Investigators and the Trial Statistician on the basis of the statistical analysis performed by the trial statistician. A draft manuscript will be submitted for review to all co-authors. Authors of the main manuscript will include the Trial Chairperson (first author), the Co-Chairperson, the Principal Investigators, the members of the steering committee, and the trial statistician. Others who have made a significant contribution to the trial may also be included as author, or otherwise will be included in the acknowledgement.

Authors of correlative manuscripts (e.g. results of translational research) will include the Trial Chairperson, the Co-Chairperson, the Principal Investigators, and those persons who have made a significant contribution to the published results.

Interim publications or presentations of the study may include demographic data, overall results and prognostic factor analyses, results for secondary endpoints, but no comparisons between randomized treatment arms for the primary endpoint may be made publicly available before the recruitment is discontinued.

Any publication, abstract or presentation based on patients included in this study must be approved by the Trial Chairperson and the Co-Chairperson. This is applicable to any individual patient or any subgroup of the trial patients. Such a publication cannot include any comparisons between randomized treatment arms or an analysis of any of the study endpoints unless the final results of the trial have already been published.

19 CONFIDENTIALITY

19.1 Copyright

The information contained in this protocol is copyright protected by the sponsor. This information is given for the needs of the trial and must not be disclosed to persons outside of the trial without prior written consent of the steering committee.

19.2 Confidentiality

Trial-related data of the patient will be provided in a codified manner to the CTU. A sequential unique patient number (UPN) will be attributed to each patient registered into the trial. Identification of patients must be guaranteed at the center. In order to avoid identification errors, a center specific identification number and the UPN have to be provided on the eCRF. Use the patient screening, enrollment and identification list. Patient confidentiality will be maintained according to applicable legislation. Patients must be informed of, and agree to, data and material transfer and handling, in accordance with Swiss data protection law. All information concerning the trial drugs supplied by Baxter SA and Robapharm/Pierre Fabre SA in connection with this trial and not previously published is considered confidential and proprietary information.

20 REFERENCES

1. Socie G, Clift RA, Blaise D, Devergie A, Ringden O, Martin PJ, et al. Busulfan plus cyclophosphamide compared with total-body irradiation plus cyclophosphamide before marrow transplantation for myeloid leukemia: long-term follow-up of 4 randomized studies. Blood. 2001 Dec 15;98(13):3569-74. PubMed PMID: 11739158. Epub 2001/12/12. eng.

2. Hassan M. The role of busulfan in bone marrow transplantation. Med Oncol. 1999 Sep;16(3):166-76. PubMed PMID: 10523796. Epub 1999/10/19. eng.

3. Clift RA, Buckner CD, Thomas ED, Bensinger WI, Bowden R, Bryant E, et al. Marrow transplantation for chronic myeloid leukemia: a randomized study comparing cyclophosphamide and total body irradiation with busulfan and cyclophosphamide. Blood. 1994 Sep 15;84(6):2036-43. PubMed PMID: 8081005. Epub 1994/09/15. eng.

4. Brodsky R, Topolsky D, Crilley P, Bulova S, Brodsky I. Frequency of veno-occlusive disease of the liver in bone marrow transplantation with a modified busulfan/cyclophosphamide preparative regimen. Am J Clin Oncol. 1990 Jun;13(3):221-5. PubMed PMID: 2346127. Epub 1990/06/01. eng.

5. Vassal G, Hartmann O, Benhamou E. Busulfan and veno-occlusive disease of the liver. Ann Intern Med. 1990 Jun 1;112(11):881. PubMed PMID: 2344115. Epub 1990/06/01. eng.

6. Ljungman P, Hassan M, Bekassy AN, Ringden O, Oberg G. High busulfan concentrations are associated with increased transplant-related mortality in allogeneic bone marrow transplant patients. Bone Marrow Transplant. 1997 Dec;20(11):909-13. PubMed PMID: 9422468. Epub 1998/01/09. eng.

7. McCune JS, Batchelder A, Deeg HJ, Gooley T, Cole S, Phillips B, et al. Cyclophosphamide following targeted oral busulfan as conditioning for hematopoietic cell transplantation: pharmacokinetics, liver toxicity, and mortality. Biol Blood Marrow Transplant. 2007 Jul;13(7):853-62. PubMed PMID: 17580264. Epub 2007/06/21. eng.

8. Busilvex®, Fachinformation des Arzneimittel-Kompendiums der Schweiz (http://www.kompendium.ch) [Internet].

9. Endoxan®, Fachinformation des Arzneimittel-Kompendiums der Schweiz (http://www.kompendium.ch) [Internet].

10. Buggia I, Zecca M, Alessandrino EP, Locatelli F, Rosti G, Bosi A, et al. Itraconazole can increase systemic exposure to busulfan in patients given bone marrow transplantation. GITMO (Gruppo Italiano Trapianto di Midollo Osseo). Anticancer Res. 1996 Jul-Aug;16(4A):2083-8. PubMed PMID: 8712747. Epub 1996/07/01. eng.

11. Meresse V, Hartmann O, Vassal G, Benhamou E, Valteau-Couanet D, Brugieres L, et al. Risk factors for hepatic veno-occlusive disease after high-dose busulfan-containing regimens followed by autologous bone marrow transplantation: a study in 136 children. Bone Marrow Transplant. 1992 Aug;10(2):135-41. PubMed PMID: 1525602.

12. Nilsson C, Aschan J, Hentschke P, Ringden O, Ljungman P, Hassan M. The effect of metronidazole on busulfan pharmacokinetics in patients undergoing hematopoietic stem cell transplantation. Bone Marrow Transplant. 2003 Mar;31(6):429-35. PubMed PMID: 12665836. Epub 2003/04/01. eng.

13. McDonald GB, Slattery JT, Bouvier ME, Ren S, Batchelder AL, Kalhorn TF, et al. Cyclophosphamide metabolism, liver toxicity, and mortality following hematopoietic stem cell transplantation. Blood. 2003 Mar 1;101(5):2043-8. PubMed PMID: 12406916. Epub 2002/10/31. eng.

14. Hassan M, Ljungman P, Ringden O, Hassan Z, Oberg G, Nilsson C, et al. The effect of busulphan on the pharmacokinetics of cyclophosphamide and its 4-hydroxy metabolite: time interval influence on therapeutic efficacy and therapy-related toxicity. Bone Marrow Transplant. 2000 May;25(9):915-24. PubMed PMID: 10800057.

15. DeLeve LD, Wang X. Role of oxidative stress and glutathione in busulfan toxicity in cultured murine hepatocytes. Pharmacology. 2000 Apr;60(3):143-54. PubMed PMID: 10754451.

16. Hassan Z, Hellstrom-Lindberg E, Alsadi S, Edgren M, Hagglund H, Hassan M. The effect of modulation of glutathione cellular content on busulphan-induced cytotoxicity on hematopoietic cells in vitro and in vivo. Bone Marrow Transplant. 2002 Aug;30(3):141-7. PubMed PMID: 12189531.

17. Nilsson C, Forsman J, Hassan Z, Abedi-Valugerdi M, O'Connor C, Concha H, et al. Effect of altering administration order of busulphan and cyclophosphamide on the myeloablative and immunosuppressive properties of the conditioning regimen in mice. Exp Hematol. 2005 Mar;33(3):380-7. PubMed PMID: 15730862. Epub 2005/02/26. eng.

18. Ferrara JL. The cytokine modulation of acute graft-versus-host disease. Bone Marrow Transplant. 1998 Jun;21 Suppl 3:S13-5. PubMed PMID: 9712485. Epub 1998/08/26. eng.

19. Hill GR, Teshima T, Rebel VI, Krijanovski OI, Cooke KR, Brinson YS, et al. The p55 TNF-alpha receptor plays a critical role in T cell alloreactivity. J Immunol. 2000 Jan 15;164(2):656-63. PubMed PMID: 10623807. Epub 2000/01/07. eng.

20. Via CS, Finkelman FD. Critical role of interleukin-2 in the development of acute graft-versus-host disease. Int Immunol. 1993 Jun;5(6):565-72. PubMed PMID: 8102248. Epub 1993/06/01. eng.

21. Sadeghi B, Jansson M, Hassan Z, Mints M, Hagglund H, Abedi-Valugerdi M, et al. The effect of administration order of BU and CY on engraftment and toxicity in HSCT mouse model. Bone Marrow Transplant. 2008 May;41(10):895-904. PubMed PMID: 18223695.

22. Cantoni N, Gerull S, Heim D, Halter J, Bucher C, Buser A, et al. Order of application and liver toxicity in patients given BU and CY containing conditioning regimens for allogeneic hematopoietic SCT. Bone marrow transplantation. 2010 Jun 14. PubMed PMID: 20548339. Epub 2010/06/16. Eng.

23. Milanowska K, Krwawicz J, Papaj G, Kosinski J, Poleszak K, Lesiak J, et al. REPAIRtoire--a database of DNA repair pathways. Nucleic acids research. 2011 Jan;39(Database issue):D788-92. PubMed PMID: 21051355. Pubmed Central PMCID: 3013684. Epub 2010/11/06. eng.

24. Manning F, Conway A, Doyle S. Differential reactivity of native and recombinant pi GST in various assay systems. Biochem Soc Trans. 1995 May;23(2):360S. PubMed PMID: 7672389. Epub 1995/05/01. eng.

25. SUVA Schweizerische Unfallversicherungsanstalt: Versicherung. Umgang mit Zytostatika. 2004.

26. Oken MM, Creech RH, Tormey DC, Horton J, Davis TE, McFadden ET, et al. Toxicity and response criteria of the Eastern Cooperative Oncology Group. American journal of clinical oncology. 1982 Dec;5(6):649-55. PubMed PMID: 7165009. Epub 1982/12/01. eng.

27. Thomas E, Storb R, Clift RA, Fefer A, Johnson FL, Neiman PE, et al. Bone-marrow transplantation (first of two parts). N Engl J Med. 1975 Apr 17;292(16):832-43. PubMed PMID: 234595. Epub 1975/04/17. eng.

28. Deeg HJ, Storb R. Graft-versus-host disease: pathophysiological and clinical aspects. Annu Rev Med. 1984;35:11-24. PubMed PMID: 6372650. Epub 1984/01/01. eng.

29. Busilvex®, Product information of the European Medicines Agency (EMEA). (http://www.ema.europa.eu/docs/en_GB/document_library/EPAR_-_Product_Information/human/000472/WC500052066.pdf).

30. (FDA) FaDA. Guidance for Clinical Trial Sponsors - Establishment and Operation of Clinical Trial Data Monitoring Committees. 2006.

31. Bauer DF. Constructing confidence sets using rank statistics. J Am Stat Assoc. 1972 (67):687-90.

32. Davison AC, Hinkley DV. Bootstrap methods and their application. Press CU, editor1987.

33. Verordnung über klinische Versuche mit Heilmitteln (VKlin) vom 17. Oktober 2001 (Stand am 1. Januar 2008) / Ordonnance sur les essais cliniques de produits thérapeutiques (OClin) du 1 janvier 2008. (http://www.admin.ch/ch/d/sr/8/812.214.2.de.pdf).

34. Heilmittelgesetz, HMG Bundesgesetz über Arzneimittel und Medizinprodukte (Heilmittelgesetz, HMG) vom 15. Dezember 2000 / Loi fédérale sur les médicaments et les dispositifs médicaux (Loi sur les produits thérapeutiques, LPT) du 15 décembre 2000. (http://www.admin.ch/ch/d/sr/8/812.21.de.pdf).

35. Declaration of Helsinki (as amended in Tokyo, Venice, Hong Kong, Somerset West, Edinburgh and clarified in Washington and Tokyo). 2004, October. http://www.wma.net/e/policy/b3.htm.

36. International Conference on Harmonization (ICH) E 6 Guideline for Good Clinical Practice. 1996. (http://www.ich.org/LOB/media/MEDIA482.pdf).

37. Verordnung vom 18. Mai 2005 über die Gute Laborpraxis (GLPV). 18. Mai 2005. http://www.admin.ch/ch/d/sr/c813_112_1.html.

38. Siekmann L, Bonora R, Burtis CA, Ceriotti F, Clerc-Renaud P, Ferard G, et al. IFCC primary reference procedures for the measurement of catalytic activity concentrations of enzymes at 37 degrees C. Part 1. The concept of reference procedures for the measurement of catalytic activity concentrations of enzymes. Clin Chem Lab Med. 2002 Jun;40(6):631-4. PubMed PMID: 12211661. Epub 2002/09/05. eng.

39. Biobanks: Obtainment, preservation and utilisation of human biological material. Medical-ethical guidelines and recommendations. SAMW guidelines 2006.

40. Tsakiris DA, Tichelli A. Thrombotic complications after haematopoietic stem cell transplantation: early and late effects. Best Pract Res Clin Haematol. 2009 Mar;22(1):137-45. PubMed PMID: 19285280. Epub 2009/03/17. eng.

41. Gratwohl A, Stern M, Brand R, Apperley J, Baldomero H, de Witte T, et al. Risk score for outcome after allogeneic hematopoietic stem cell transplantation: a retrospective analysis. Cancer. 2009 Oct 15;115(20):4715-26. PubMed PMID: 19642176. Epub 2009/07/31. eng.

42. Sorror ML, Maris MB, Storb R, Baron F, Sandmaier BM, Maloney DG, et al. Hematopoietic cell transplantation (HCT)-specific comorbidity index: a new tool for risk assessment before allogeneic HCT. Blood. 2005 Oct 15;106(8):2912-9. PubMed PMID: 15994282. Pubmed Central PMCID: 1895304. Epub 2005/07/05. eng.

43. Bearman SI. The syndrome of hepatic veno-occlusive disease after marrow transplantation. Blood. 1995 Jun 1;85(11):3005-20. PubMed PMID: 7756636. Epub 1995/06/01. eng.

Appendix 1A Schedule of treatments

Group A, standard group

Tag	Konditionierung
	**Start Hydrierung** 12 Stunden vor Beginn Konditionierung
-8	**Busilvex®** 0.8 mg/kg/6-stdl. in NaCl 0.9% i.v. über 2 h (Total 16 Dosen)
-7	
-6	
-5	
-4	
	**Mind. 24 Std. Abstand zwischen der letzten BU-Dosis und der ersten CY-Dosis**
-3	**Endoxan®** 60 mg/kg/Tag in 500 ml Gluc 5% i.v. über 1 h
-2	**Endoxan®** 60 mg/kg/Tag in 500 ml Gluc 5% i.v. über 1 h
-1	**Mind. 24 Std. Abstand zwischen der letzten CY-Dosis und HSZT**
0	**HSZT**
1	**MTX** 15 mg/m2/Tag in 100 ml NaCl 0.9% i.v. über 15 Min. nur Pat. > 20 J.
2	**Leukovorin** gem. Schema der Klinik
3	**MTX** 10 mg/m2/Tag in 100 ml NaCl 0.9% i.v. über 15 Min. nur Pat. > 20 J.
4	**Leukovorin** 15 mg i.v. je 24 h und 30 h nach MTX-Applikation **Leukovorin** gem. Schema der Klinik
5	
6	**MTX** 10 mg/m2/Tag in 100 ml NaCl 0.9% i.v. über 15 Min. nur Pat. > 20 J.
7	**Leukovorin** gem. Schema der Klinik

	Zusätzlich
	**Uromitexan** Amp. mg i.v. an Endoxan-Tagen:<br>20% der Endoxan-Dosis **Stunde –1; +4; +8** sowie<br>10% der Endoxan-Dosis **Stunde +14 und 6-stdl.** bis und mit **Tag -1**
	**Temesta** exp. 3 x 1 mg / Tag p.os **Beginn 12 Stunden vor der ersten BU-Dosis bis 24 Stunden nach der letzten BU-Dosis**
	**CYA** Amp. i.v. in 250 Misch über 6 h **ab Tag –3**

Group B, experimental group

Tag	Konditionierung
	**Start Hydrierung** 12 Stunden vor Beginn Konditionierung
-8	**Endoxan®** 60 mg/kg/Tag in 500 ml Gluc 5% i.v. über 1 h
-7	**Endoxan®** 60 mg/kg/Tag in 500 ml Gluc 5% i.v. über 1 h
	**Mind. 24 Std. Abstand zwischen der letzten CY-Dosis und der ersten BU-Dosis**
-6	
-5	
-4	**Busilvex®** 0.8 mg/kg/6-stdl. in NaCl 0.9% i.v. über 2 h (Total 16 Dosen)
-3	
-2	
-1	**Mind. 24 Std. Abstand zwischen der letzten BU-Dosis und HSZT**
0	**HSZT**
1	**MTX** 15 mg/m2/Tag in 100 ml NaCl 0.9% i.v. über 15 Min. nur Pat. > 20 J.
2	**Leukovorin** gem. Schema der Klinik
3	**MTX** 10 mg/m2/Tag in 100 ml NaCl 0.9% i.v. über 15 Min. nur Pat. > 20 J.
4	**Leukovorin** gem. Schema der Klinik
5	
6	**MTX** 10 mg/m2/Tag in 100 ml NaCl 0.9% i.v. über 15 Min. nur Pat. > 20 J.
7	**Leukovorin** gem. Schema der Klinik

	Zusätzlich
	**Uromitexan** Amp. mg i.v. an Endoxan-Tagen:<br>20% der Endoxan-Dosis **Stunde –1; +4; +8** sowie<br>10% der Endoxan-Dosis **Stunde +14 und 6-stdl.** bis und mit **Tag -5**
	**Temesta** exp. 3 x 1 mg / Tag p.os **Beginn 12 Stunden vor der ersten BU-Dosis bis 24 Stunden nach der letzten BU-Dosis**
	**CYA** Amp. i.v. in 250 Misch über 6 h **ab Tag –3**

Conditioning regimen: treatment schedule

**Group A**	**Group B**
d-8 BU 17:00 (1)<br>BU 23:00 (2)<br>d-7 BU 5:00 (3)<br>BU 11:00 (4)<br>BU 17:00 (5)<br>BU 23:00 (6)<br>d-6 BU 5:00 (7)<br>BU 11:00 (8)<br>BU 17:00 (9)<br>BU 23:00 (10)<br>d-5 BU 5:00 (11)<br>BU 11:00 (12)<br>BU 17:00 (13)<br>BU 23:00 (14)<br>d-4 BU 5:00 (15)<br>BU 11:00 (16)<br>Interval between last Bu and first CY therapy has to be at least 24h!<br>d-3 CY 13:00 (1)<br>d-2 CY 13:00 (2)<br>Interval between last chemotherapy and HSCT has to be at least 24h!<br>d 0 HSZT 14:00	d-8 CY 13:00 (1)<br>d-7 CY 13:00 (2)<br>Interval between last CY and first BU therapy has to be at least 24h!<br>d-6 BU 17:00 (1)<br>BU 23:00 (2)<br>d-5 BU 5:00 (3)<br>BU 11:00 (4)<br>BU 17:00 (5)<br>BU 23:00 (6)<br>d-4 BU 5:00 (7)<br>BU 11:00 (8)<br>BU 17:00 (9)<br>BU 23:00 (10)<br>d-3 BU 5:00 (11)<br>BU 11:00 (12)<br>BU 17:00 (13)<br>BU 23:00 (14)<br>d-2 BU 5:00 (15)<br>BU 11:00 (16)<br>Interval between last chemotherapy and HSCT has to be at least 24h!<br>d 0 HSZT 14:00

Appendix 1B Supportive Care

VOD prophylaxis

All the patients will receive a VOD prophylaxis with

i) Heparin intravenous 5000 IU/24h

ii) Ursodeoxycholic acid per oral 250 mg three times daily.

The VOD prophylaxis can be stopped after stable neutrophil engraftment (ANC > 0.5 x 10e9/l).

In patients with high risk for VOD an additional treatment with defribrotide(40) according to the institutional guidelines of the different centers will be performed.. High risk for VOD is defined by the presence of 1 risk factor (see below)

Risk factors for VOD

- Pre-existing hepatic damage (defined as ASAT/ALAT > 2x ULN) before starting the conditioning regimen
- Pre-existing hepatic disease (e.g. hepatitis, hemochromatosis, fibrosis, cirrhosis) before starting the conditioning regimen
- Previous hepatic irradiation
- Advanced disease (≥ CR2 for acute leukemia or several lines of treatment for other diseases)
- ≥ 2 previous HSCT
- Previous therapy with Mylotarg®
- Unrelated donor
- Patient or donor with positive CMV serology

VOD treatment (40)

The VOD treatment will be performed according to the institutional guidelines of the different centers. Details will be documented on the eCRF.

GVHD prophylaxis

The GVHD prophylaxis is identical in both cohorts and consisted of

i) Cyclosporine A: intravenous 3 mg/kg once daily or per oral 6 mg/kg daily divided in two dose) from day -3

ii) Intravenous methotrexate: 10 mg/m2 on day 1 and 6 mg/m2 on day 3 and 6. A rescue therapy with leucovorin intravenous 15 mg 24 and 30 hours after application of methotrexate will be also performed.

iii) An additional GVHD prophylaxis (e.g. ATG) can be performed according to the institutional guidelines of the different centers. Details will be documented on the CRF (MED-A Form).

Prophylaxis of central nervous system adverse reactions

All the patients will receive a prophylaxis for BU-related central nervous system adverse reactions with

i) Lorazepam: 1 mg per oral three times daily started 12 hours before the first BU dose and stopped 24 hours after the last BU dose.

Prophylaxis of hemorrhagic cystitis

To prevent a hemorrhagic cystitis after application of CY patients will receive

i) Hyperhydration: Patients will be hydrated with D5’NS (glucose 5% NaCl 0.45% + 20 mEq KCl/l + 5 mg Furosemide/l) iv at 200 ml/hr for 72 hrs beginning 2 hrs before the first CY dose. KCl will be further supplemented in case of hypokalaemia. An average urinary flow of at least 100 ml/hr will be maintained during 48 hrs following the beginning of the CY infusion. Diuretics (e.g. furosemide) will be added during this period depending on fluid in- and output status.

iii) Sodium bicarbonate for urine alkalinization can be accessorily administrated: e.g. NaBic 8.4% intravenous 100 ml at the days with CY application and the following day (from day -3 resp. day -7 until day -1 resp. day -5 in the group A resp. B).

iv) Uromitexan 300 mg/m2 will be administered at -10 min prior to CY infusion, + 4 hrs, +8 hours and +12 hours following CY infusion on days -3 and -2 in the group A resp. on days -7 and -6 in the group B.

Antiemesis

Before Busulfan and Cyclophosphamide infusions, patients will be premedicated with antiemetics according to the institutional guidelines of the different centers.

Allogeneic SCT

Allogeneic SCT will be carried out according to the standard guidelines and general operational procedures in the local allogeneic bone marrow transplantation centers. Other basic transplant strategies as well as supportive care measures are performed according to the institutional guidelines of the different centers. Details will be documented on the CRF (MED-A Form).

Special management orders

All men and pre-menopausal women should use adequate contraception during the study. Sperm should be frozen before the start of treatment from men who wish to have children.

Appendix 2 Karnofsky performance status(26)

100	Normal no complaints; no evidence of disease.
90	Able to carry on normal activity; minor signs or symptoms of disease.
80	Normal activity with effort; some signs or symptoms of disease.
70	Cares for self; unable to carry on normal activity or to do active work.
60	Requires occasional assistance, but is able to care for most of his personal needs.
50	Requires considerable assistance and frequent medical care.
40	Disabled; requires special care and assistance.
30	Severely disabled; hospital admission is indicated although death not imminent.
20	Very sick; hospital admission necessary; active supportive treatment necessary.
10	Moribund; fatal processes progressing rapidly.
0	Dead

Appendix 3 European Group for Blood and Marrow Transplantation (EBMT) risk score and Sorror comorbidity index

A) EBMT risk score (41)

Risk Factor	Score Point
Age of the patient, y	
<20	0
20-40	1
>40	2
Disease stage[1]	
Early	0
Intermediate	1
Late	2
Time interval from diagnosis to transplant[2]	
<12 months	0
>12 months	1
Donor type	
HLA[3]-identical sibling donor	0
Unrelated donor	1
Donor-recipient sex combination	
All other	0
Donor female, male recipient	1

[1] See text for the definitions according to main disease category; does not apply for patients with severe aplastic anemia (score 0).

[2] Does not apply for patients transplanted in first complete remission (score 0).

[3] HLA indicates human leukocyte antigen.

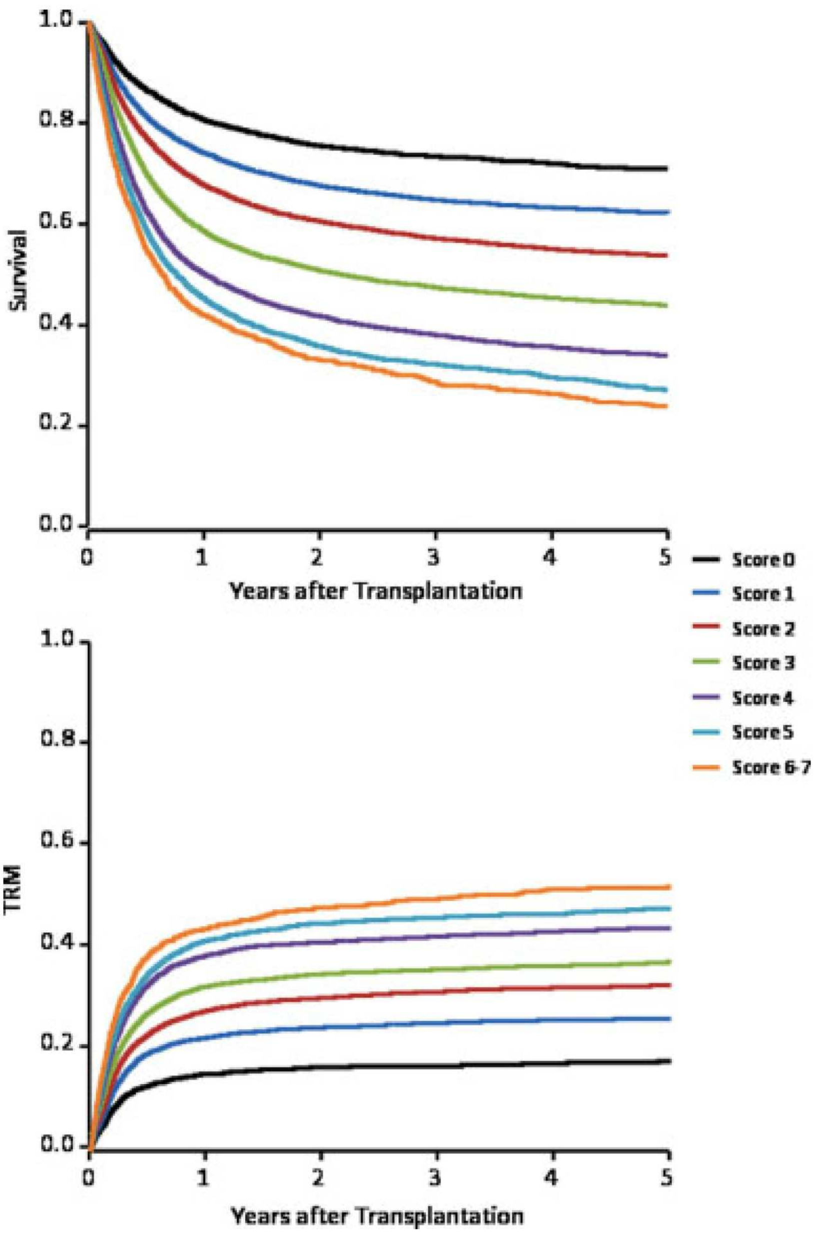

Figure 1 - Survival and transplant-related mortality (TRM) of 56,605 patients with an allogeneic hematopoietic stem cell transplantation (HSCT) for an acquired hematological disorder is shown by EBMT risk score. Graphs reflect probability of survival and transplant-related mortality over the first 5 years after HSCT.(41)

B) Sorror comorbidity index (HSCT comorbidity index) (42)

Comorbidity	Definitions of comorbidities	HSCT-CI weighted scores
Arrhythmia	Atrial fibrillation or flutter, sick sinus syndrome, or ventricular arrhythmias	1
Cardiac	Coronary artery disease, congestive heart failure, myocardial infarction, or EF50%	1
Inflammatory bowel disease	Crohn disease or ulcerative colitis	1
Diabetes	Requiring treatment with insulin or oral hypoglycemics but not diet alone	1
Cerebrovascular disease	Transient ischemic attack or cerebrovascular accident	1
Psychiatric disturbance	Depression or anxiety requiring psychiatric consult or treatment	1
Hepatic, mild	Chronic hepatitis, bilirubin > ULN to 1.5 x ULN , or AST/ALT > ULN to 2.5 x ULN	1
Obesity	Patients with a body mass index > 35 kg/m2	1
Infection	Requiring continuation of antimicrobial treatment after day 0	1
Rheumatologic	SLE, RA, polymyositis, mixed CTD, or polymyalgia rheumatica	2
Peptic ulcer	Requiring treatment	2
Moderate/severe renal	Serum creatinine > 2 mg/dL *, on dialysis, or prior renal transplantation	2
Moderate pulmonary	DLco and/or FEV1 66%-80% or dyspnea on slight activity	2
Prior solid tumor	Treated at any time point in the patient's past history, excluding nonmelanoma skin cancer	3
Heart valve disease	Except mitral valve prolapse	3
Severe pulmonary	DLco and/or FEV1 65% or dyspnea at rest or requiring oxygen	3
Moderate/severe Hepatic	Liver cirrhosis, bilirubin > 1.5 x ULN, or AST/ALT > 2.5 x ULN	3

* To convert creatinine from milligrams per deciliter to micromoles per liter, multiply milligrams per deciliter by 88.4.

EF indicates ejection fraction; ULN, upper limit of normal; SLE, systemic lupus erythmatosis; RA, rheumatoid arthritis; CTD, connective tissue disease; DLco, diffusion capacity of carbon monoxide; CI, comorbidity index

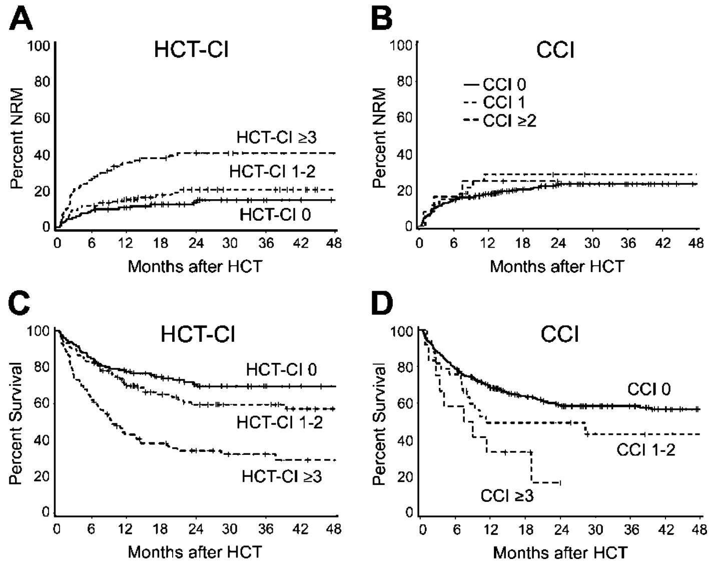

Figure 1 - The HCT-CI compared with the CCI. Cumulative incidences of nonre- lapse mortality (NRM) as stratified by the (A) new HCT-CI compared with (B) the original CCI, and Kaplan-Meier estimates of survival as stratified by (C) the new HCT-CI compared with (D) the original CCI among patients of the validation set. Only 13% of patients had scores of 1 or more when scored by the original CCI compared with 62% when scored by the new HCT-CI.

Appendix 4 Veno-occlusive disease (VOD)(43)

The diagnosis of VOD is based on the presence of two of the three clinical manifestations according to the Seattle criteria:

i) Hyperbilirubinaemia and

ii) Painful hepatomegaly or

iii) Fluid retention

Appendix 5 Graft versus Host Disease (GVHD)(27, 28)

Severity and organ involvement of acute GVHD will be assessed daily during the hospitalization and weekly for outpatients according to established criteria. Whenever possible, diagnosis of GVHD should be confirmed by skin or gut biopsy.Clinical staging of acute GVHD:

Stage	Skin	Liver (bilirubin level)	Gut
**0**	No rash	<34 umol/l	Diarrhea <500 ml/day
**+**	Maculopapular rash on <25% of body surface	34-52 umol/l	Diarrhea 500-1000 ml/day or persistent nausea
**++**	Maculopapular rash on 25-50% of body surface	53-103 umol/l	Diarrhea 1000-1500 ml/day
**+++**	Generalized erythroderma	104-256 umol/l	Diarrhea >1500 ml/day
**++++**	Generalized erythroderma with Desquamation and bullae	> 256 umol/l	Pain with or without ileus

Clinical grading of acute GVHD

Overall Grade	Stage			
	**Skin**	**Liver**	**Gut**	**Functional Impairment**
**0**	0	0	0	0
**I**	+ to ++	0	0	0
**II**	+ to +++	+	+	+
**III**	++ to +++	++ to +++	++ to +++	++
**IV**	++ to ++++	++ to ++++	++ to ++++	+++

Appendix 6 Assessment of the liver function

Liver function will be measured at the local laboratory by routine photometric assay (in accordance with the procedures of the International Federation of Clinical Chemistry, IFCC)(38) for levels of bilirubin and liver enzymes (aspartate amino transferase [AST], alanine amino transferase [ALT], gamma glutamyl transpeptidase [GGT] and alkaline phosphatase [AP]) at:

i) Day -8 (before starting the conditioning regimen)

ii) Day 0 (day of transplantation)

iii) Day +10, +20, +30, and +100

iv) Other measurement according to the institutional guidelines of the different centers.

These values are documented on the eCRF.

Appendix 7 Principal investigator’s agreement

Cyclophosphamide-Busulfan versus Busulfan-Cyclophosphamide as Conditioning Regimen before Allogeneic Hematopoietic Stem Cell Transplantation for Leukemia: a Prospective Randomized Study to Assess Liver Toxicity.

I have thoroughly read the above-mentioned trial protocol.

Having read and understood the requirements and conditions of the trial protocol, I agree to conduct the trial as specified in the protocol.

I agree to perform the clinical trial according to the international good clinical practice principles (ICH-GCP), the Declaration of Helsinki and the “Verordnung über klinische Versuche mit Heilmitteln/Ordonnance sur les essais cliniques de produits thérapeutiques”.

I confirm that there are adequate resources at my institution to conduct the trial according to protocol and submit data in a timely fashion.

I agree to adequately train sub-investigators, clinical research personnel and further persons involved in the conduct of the trial.

I agree to keep a list in the investigator site file of appropriately qualified persons to whom I have delegated significant trial-related duties.

I agree to ask for approval from the relevant ethics committee for the protocol, patient information and any amendment, which needs approval. For administrative amendments not requiring a formal approval, I will obtain a letter of receipt from the ethics committee. I will inform the CTU in case the ethics committee withdraws approval of the trial.

I will only recruit patients into this trial once the IEC and, if applicable, Swissmedic has approved the center.

I agree to use the trial material, including medication, only as specified in the protocol.

I agree to appropriately inform patients on the trial including risks, possible adverse drug reactions and means how to reduce/treat side effects. Informed consent will be obtained and signed from each patient prior to enrollment and prior to application of any trial-specific procedure.

I understand that changes to the protocol must be made in the form of an amendment, which has the prior written approval of the steering committee and the relevant regulatory bodies.

I understand that any violation of the protocol may lead to early termination of the trial at my institution.

I agree to report to the CTU and the relevant ethics committee, within one working day, any clinical adverse event that is serious (SAE) and Serious Adverse Drug Reactions (SADRs), whether considered treatment-related or not. Follow-up reports will be provided within the timelines specified on the SAE Form.

I agree to keep accurate records on all patient information (case report forms and patient informed consent statement), and all other information collected during the trial for a minimum period of 10 years after termination of the trial.

I will provide the required documents and information for monitors and all people responsible for auditing/inspecting.

I confirm to be present at the center and responsible for the whole trial period. In case I leave the center, I will take care to hand over the responsibility of the principal investigator at the center to somebody else and I will inform the CTU and the ethics committee accordingly.

I agree not to close the trial at my center without prior written information to the CTU.

I agree not to publicize all or any part of the results of the trial carried out under this protocol before primary publication in a peer-reviewed journal, and I agree to notify the steering committee of any planned publications thereafter.

I will take care that all members of the local trial team will comply with the content of this agreement.

Principal investigator:

Name:____ Title:________________

Center:____

Date:_______________________ Signature:_______________________________________

The signed original of this agreement has to be sent to the CTU. A copy of this page has to be submitted to your local ethics committee and another has to be stored in your investigator site file.

Appendix 8 Translational research analyses

Procedure for cytokines profiling

Handling and processing of samples

- For cytokines profiling, one blood samples (in Serum tubes) at each time (day 0, day 10, day 20, day 30) will be collected either by direct venipuncture, via indwelling cannula inserted in a forearm vein, or via an indwelling intravenous access device such as a Broviac catheter or a Port-à-Cath according to the Appendix 9 "Checkliste for collection of samples for pharmacogenomics and cytokines". No tubes will be provided by the sponsor, all the centers have to use their own material.

Plasma banking

Plasma aliquots, which are not used for the cytokines profiling will be banked (plasma banking) and kept for 20 years for future analysis in connection with cytokines and conditioning regimen after allogeneic HSCT (see also 18.8). The plasma banking will be done at the University Hospital of Basel, Basel, Switzerland.

Samples shipping

If necessary (Geneva and Zürich), the samples will be collected *at the end of the study* and transported to the laboratory in Basel for the cytokines profiling.

The shipping will always organized by the CTU Basel. Samples will be transported on dry ice.

Procedure for genetic polymorphism screening/analysis

Handling and processing of samples

For genetic polymorphism analysis blood samples (in EDTA tubes) will be collected either by direct venipuncture, via indwelling cannula inserted in a forearm vein, or via an indwelling intravenous access device such as a Broviac catheter or a Port-à-Cath according to the Appendix 9 "Checkliste for collection of samples for pharmacogenomics and cytokines".

No tubes will be provided by the sponsor, all the centers have to use their own material.

Samples shipping

If necessary (Basel and Zürich), the samples will be shipped *at the end of the study* to the laboratory in Geneva for the DNA analysis. The shipping will be organized by the CTU Basel.

DNA banking

The genetic polymorphism analysis and DNA banking (see also 14.3) will be done at the HUG Geneva University Hospital, Geneva, Switzerland.

Expression analysis of BER and GST pathway genes

Handling and processing of samples

For expression analysis of BER and GST pathway genes blood samples (in PAXgene® Blood RNA tube) tubes) will be collected either by direct venipuncture, via indwelling cannula inserted in a forearm vein, or via an indwelling intravenous access device such as a Broviac catheter or a Port-à-Cath according to the Appendix 9 “Checkliste for collection of samples for pharmacogenomics and cytokines”.

No tubes will be provided by the sponsor, all the centers have to use their own material.

Samples shipping

If necessary (Basel and Zürich), the samples will be shipped *at the end of the study* to the laboratory in Geneva for the pathway analysis. The shipping will be organized by the CTU Basel.

Procedure for alpha GST levels analysis and glutathione analysis

Handling and processing of samples

For alpha GST levels analysis blood samples (in EDTA tubes) will be collected either by direct venipuncture, via indwelling cannula inserted in a forearm vein, or via an indwelling intravenous access device such as a Broviac catheter or a Port-à-Cath according to the Appendix 9 “Checkliste for collection of samples for pharmacogenomics and cytokines”.

No tubes will be provided by the sponsor, all the centers have to use their own material.

Samples shipping

If necessary (Basel and Zürich), the samples will be shipped *at the end of the study* to the laboratory in Basel for the alpha GST level analysis. The shipping will be organized by the CTU Basel.

Appendix 9 Checklist for collection of samples for pharmacogenomics and pharmacokinetics

Group A

Date and Time	Sample Nr.	Samples to be collected	Sample	Sample ID (followed by "Group A/B" and "patient UPN")	Purpose	Check (Yes/No) and remarks
**Day -8** (before Bu administration)	1	**5mL (vial 1 EDTA)**	Plasma	**GSTA0**	alpha GST and Glutathion levels	
		**5mL (vial 2 EDTA)**	DNA	**DNA0**	DNA analysis/banking	
		**2x2.5mL (2 PAXgene® Blood RNA tubes)**	RNA	**RNA0**	RNA analysis/banking	
		**8.5mL (Serum)**	Serum	**Cyto0**	Cytokines level	
**Day -3** (before Cy administration)	2	**5mL (EDTA)**	Plasma	**GSTA1**	alpha GST and Glutathion levels	
		**2x2.5mL (2 PAXgene® Blood RNA tubes)**	RNA	**RNA1**	RNA analysis/banking	
**Day 0**	3	**5mL (EDTA)**	Plasma	**GSTA2**	alpha GST and Glutathion levels	
		**2x2.5mL (2 PAXgene® Blood RNA tubes)**	RNA	**RNA2**	RNA analysis/banking	
		**8.5mL (Serum)**	Serum	**Cyto2**	Cytokines level	
**Day 10**	4	**8.5mL (Serum)**	Serum	**Cyto3**	Cytokines level	
**Day 20**	5	**8.5mL (Serum)**	Serum	**Cyto4**	Cytokines level	
**Day 30**	6	**8.5mL (Serum)**	Serum	**Cyto5**	Cytokines level	

Group B

Date and Time	Sample Nr.	Samples to be collected	Sample	Sample ID (followed by "Group A/B" and "patient UPN")	Purpose	Check (Yes/No) and remarks
**Day -8** (before Cy administration)	1	**5mL (vial 1 EDTA)**	Plasma	**GSTA0**	alpha GST and Glutathion levels	
		**5mL (vial 2 EDTA)**	DNA	**DNA0**	DNA analysis/banking	
		**2x2.5mL (2 PAXgene® Blood RNA tubes)**	RNA	**RNA0**	RNA analysis/banking	
		**8.5mL (Serum)**	Serum	**Cyto0**	Cytokines level	
**Day -6** (before Bu administration)	2	**5mL (EDTA)**	Plasma	**GSTA1**	alpha GST and Glutathion levels	
		**2x2.5mL (2 PAXgene® Blood RNA tubes)**	RNA	**RNA1**	RNA analysis/banking	
**Day 0**	3	**5mL (EDTA)**	Plasma	**GSTA2**	alpha GST and Glutathion levels	
		**2x2.5mL (2 PAXgene® Blood RNA tubes)**	RNA	**RNA2**	RNA analysis/banking	
		**8.5mL (Serum)**	Serum	**Cyto2**	Cytokines level	
**Day 10**	4	**8.5mL (Serum)**	Serum	**Cyto3**	Cytokines level	
**Day 20**	5	**8.5mL (Serum)**	Serum	**Cyto4**	Cytokines level	
**Day 30**	6	**8.5mL (Serum)**	Serum	**Cyto5**	Cytokines level	

Collection of blood samples

Plasma: GSTA0 / GSTA1 / GSTA2

1. Draw blood (5 ml) directly into a tube containing EDTA using standard techniques
2. Place tube on crushed ice, immediately
3. Centrifuge at 2’000g for 10 min (cold)
4. Separate plasma without disturbing buffy coat and aliquot at 0.5 ml
5. **Store at –80°C**

DNA: DNA0

1. Draw blood (5 ml) directly into a tube containing EDTA using standard techniques
2. Store the “Vial 1 EDTA” at day -8 (see above, “**DNA0**”) direct **at –80°C**.

RNA: RNA0/RNA1/RNA2

Note: Stand the PAXgene® Blood RNA Tubes always upright in a wire rack.

Do not freeze tubes upright in a Styrofoam tray as this may cause the tubes to crack.

The frozen PAXgene® Blood RNA Tubes are subject to breakage upon impact. To reduce the risk of breakage during shipment, frozen tubes should be treated in the same manner as glass tubes.

1. Draw blood (2x2.5 ml) directly in 2 PAXgene® Blood RNA tubes using standard techniques
2. Store the PAXgene® Blood RNA Tubes at room temperature (18°C to 25°C) for a minimum of 2 hours and a maximum of 72 hours before transferring to the freezer.
3. Freeze the tubes first at **-20°C for 24 hours**, and then **transfer them to -80°C**.

Serum: Cyto0 / Cyto2 / Cyto3 / Cyto4 / Cyto5

1. Draw blood (8.5 ml) directly into a Serum tube using standard techniques
2. Clot at room temperature for at least 20 minutes but not longer than 1 hour
3. Centrifuge at 2’000 g for 10 min (room tempereture)
4. Separate serum without disturbing buffy coat and aliquot at 0.5 ml
5. **Store at –80°C**

Important notes

1. Dedicated personnel for sample management will avoid errors in the collection, labeling and storage of the samples. The tubes (incl. labeling) and the accompanying form will be prepared by a CRA.

2. Collection in EDTA, PAXgene® or Serum tubes with patient UPN, group A or B and sample ID properly labeled on each of the tube.

3. All the aliquots must be properly labeled with patient UPN, group A or B, sample ID and aliquot number.

4. Patient UPN shall be unique for each patient and will be decided by the CTU/CRA at randomization in the BuCyBu trial.

5. All samples collected must be stored at -80 ^{0}C. Shipment in dry ice only preferably should reach Geneve on Thursday (can ship on Wednesday)

6. Sample labels shall be blinded before shipping to Geneva (except the patient UPN and sample ID) to avoid bias. When the sample labels are blinded the information shall not be shared with Geneva till the end of analysis and only at the end of analysis this data must be shared (till then properly maintained by the CRA)

7. Address for shipping to Geneva or for questions:

   Marc Ansari, MD, Département de l’enfant et adolescent, Hôpital Cantonal de Genève (HUG), Bureau 1H-5-507, Rue Willy Donzé 6, 1211 Genève 14

   Phone: +41795023389, Phone: +41223824731, Fax: +41223823100

   Email: marc@CanSearCH.ch

Appendix 10 Trial overview

	Screening	Enrollment	Treatment								HSCT	Follow-up			
Day	-21	-9	-8	-7	-6	-5	-4	-3	-2	-1	0	10	20	30	100
Informed Consent	X														
Pregnancy Test		X													
Medical History		X													
Physical Examination<br>Hepatosplenomegaly<br>Karnofsky performance status		X									X			X	X
EBMT and Sorror risk scores		X													
Liver Examination (CT or Ultrasound)		X													
Randomization		X													
Treatment Group A															
Busulfan			X	X	X	X	X								
Cyclophosphamide								X	X						
Treatment Group B															
Busulfan					X	X	X	X	X						
Cyclophosphamide			X	X											
VOD											X	X	X	X	X
**Blood samples**															
Serology: HIV, Hep A/B/C, CMV, EBV (1x 5ml blood for serum separation: routine sample)	X														
Assessment of liver enzymes (1x 5ml heparin blood: routine sample)	X		X								X	X	X	X	X
Pharmacokinetics															
Group A				6X	*	*	*								
Group B						6X	*	*	*						
Pharmakogenomics<br>- DNA Analysis/Banking (day -8 only)<br>- RNA Analysis (BER/GST pathway)<br>- alpha GST level and Glutathione															
Group A (2x5ml EDTA blood and 2x2.5ml PAXgene® blood)			X					X			X				
Group B (2x5ml EDTA blood and 2x2.5ml PAXgene® blood)			X		X						X				
Cytokine profiling															
Group A (1x8.5ml Serum)			X								X	X	X	X	
Group B (1x8.5ml Serum)			X								X	X	X	X	

* Further blood analyses for the pharmacokinetics of BU are dependent on the first pharmacokinetics and are performed independently of this study and according to the institutional guidelines of the center (see Section 12.4).
